# New Polyenes from the Marine-Derived Fungus *Talaromyces cyanescens* with Anti-Neuroinflammatory and Cytotoxic Activities

**DOI:** 10.3390/molecules26040836

**Published:** 2021-02-05

**Authors:** Hee Jae Shin, Cao Van Anh, Duk-Yeon Cho, Dong-Kug Choi, Jong Soon Kang, Phan Thi Hoai Trinh, Byeoung-Kyu Choi, Hwa-Sun Lee

**Affiliations:** 1 Marine Natural Products Chemistry Laboratory, Korea Institute of Ocean Science and Technology, 385 Haeyang-ro, Yeongdo-gu, Busan 49111, Korea; caovananh@kiost.ac.kr (C.V.A.); choibk4404@kiost.ac.kr (B.-K.C.); hwasunlee@kiost.ac.kr (H.-S.L.); 2 Department of Marine Biotechnology, University of Science and Technology (UST), 217 Gajungro, Yuseong-gu, Daejeon 34113, Korea; 3 Department of Applied Life Science, Graduate school, BK21 Program, Konkuk University, Chungju 27478, Korea; whejrdus10@kku.ac.kr (D.-Y.C.); choidk@kku.ac.kr (D.-K.C.); 4 Laboratory Animal Resource Center, Korea Research Institute of Bioscience and Biotechnology, 30 Yeongudanjiro, Cheongju 28116, Korea; kanjon@kribb.re.kr; 5 Department of Marine Biotechnology, Nhatrang Institute of Technology Research and Application, Vietnam Academy of Science and Technology, 02 Hung Vuong, Nha Trang 650000, Vietnam; phanhoaitrinh84@gmail.com

**Keywords:** *Talaromyces cyanescens*, marine-derived fungus, polyenes, cytotoxicity, anti-neuroinflammatory

## Abstract

Three new polyene compounds, talacyanols A–C (**1**–**3**), along with two known compounds, ramulosin (**4**) and eurothiocin A (**5**), were isolated from the marine fungus *Talaromyces cyanescens* derived from a seaweed *Caulerpa* sp. Structures of **1**–**5** were established by one-dimensional and two-dimensional (1D/2D) NMR, HR-ESIMS, and the modified Mosher’s methods, as well as comparison with previously reported literature data. All the compounds (**1**–**5**) were tested for their in vitro cytotoxic and anti-neuroinflammatory activities. Among them, **1** showed moderate cytotoxic activity against a panel of cancer cell lines (HCT-15, NUGC-3, NCI-H23, ACHN, PC-3, and MDA-MB-231) with GI_50_ values ranging from 44.4 to 91.6 μM, whereas compounds **2** and **5** exhibited anti-neuroinflammatory effect without cytotoxicity against all the tested cell lines.

## 1. Introduction

Despite developing new therapeutic agents is a long, intricate, and costly process, the discovery and development of the new drugs are urgently needed due to the increase in the annual number of deaths caused by cancer, cardiovascular, respiratory, and neurodegenerative diseases, as well as the emergence and rapid growth of multidrug resistant pathogenic microbes [1,2].

Epiphytic and endophytic fungi have been known as potential sources of biologically active compounds, which may both directly and indirectly be used as therapeutically active substances against a wide variety of diseases [3,4,5]. Among them, the fungal genus *Talaromyces* has been recognized to be the prolific producers of structurally diverse and pharmacologically active secondary metabolites [6]. A large proportion of compounds, including alkaloids [7], terpenoids [8], polyketides [9], lactones [10], and quinones [11] demonstrated various bioactivities such as anticancer [12], antimicrobial [9], and antioxidant activities [13].

**Figure 1:**
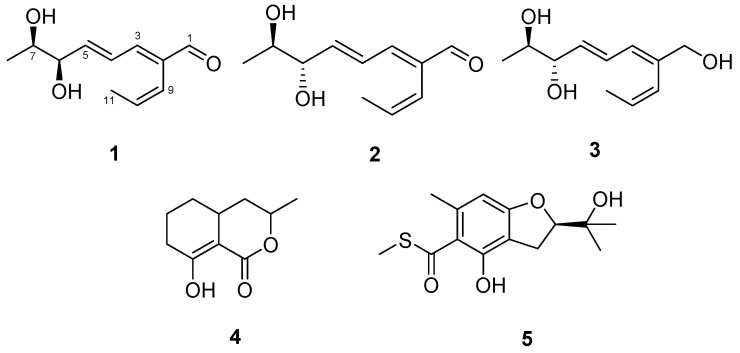
Structures of compounds **1**–**5** isolated from *Talaromyces cyanescens*.

Cancer and Alzheimer’s disease are the top leading causes of death worldwide, and neuro-inflammation plays a crucial role in the pathogenesis of Alzheimer’s disease [14,15]. Therefore, there is an urgent and continuous need to find new classes of anticancer and anti-neuroinflammatory drugs. As a part of our continuing studies for novel marine fungal agents with potent cytotoxic and anti-neuroinflammatory effects, we isolated three new compounds possessing a polyene skeleton, talacyanols A–C (**1**–**3**), and two known compounds ramulosin (**4**) and eurothiocin A (**5**) from the marine-derived fungus *Talaromyces* sp. 168ST-51.1 (Figure 1). In this paper, we describe the isolation and structure identification of the secondary metabolites **1**–**5** and their in vitro cytotoxic and anti-neuroinflammatory activities.

## 2. Results and Discussion

### 2.1. Structural Elucidation

Compound **1** was isolated as a colorless oil with a molecular formula of C_11_H_16_O_3_, which was established from an HRESIMS peak at *m*/*z* 219.1002 [M + Na]^+^ (calcd. for C_11_H_16_O_3_Na^+^, 219.0992). The ^1^H NMR spectrum displayed signals of an aldehydic proton at *δ*_H_ 9.46 (H-1), five olefinic protons at *δ*_H_ 5.93–7.13, two oxymethine protons at *δ*_H_ 3.72 (H-7) and 4.09 (H-6), and two methyl groups at *δ*_H_ 1.14 (H_3_-8) and 1.54 (H_3_-11) (Table 1). The ^13^C NMR data exhibited the presence of eleven resonances, which were assigned for an aldehyde group at *δ*_C_ 196.0 (C-1), five protonated olefinic carbons at *δ*_C_ 150.2 (C-3), 146.0 (C-5), 132.5 (C-10), 128.8 (C-4), and 122.0 (C-9), a sp^2^ quaternary carbon at *δ*_C_ 139.1 (C-2), two oxygenated methines at *δ*_C_ 77.0 (C-6), 71.4 (C-7), and two methyls at *δ*_C_ 18.7 (C-8), 15.7 (C-11) (Table 1). The above data suggested that **1** possesses an acyclic skeleton with an aldehyde and three pairs of sp^2^ carbons, accounting for all four degrees of unsaturation in accordance with its molecular formula.

**Figure 2:**
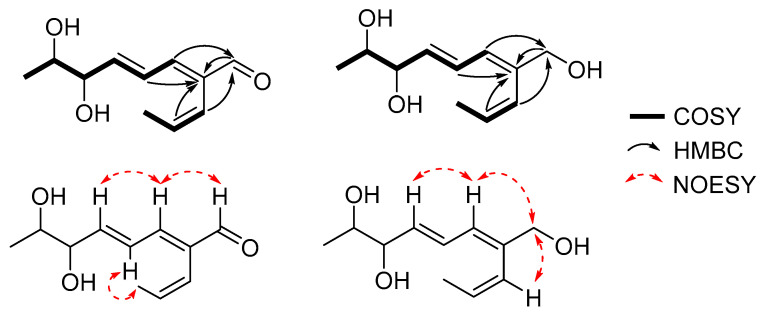
Key COSY, HMBC, and NOESY correlations for compounds **1**–**3**.

Detailed analysis of ^1^H-^1^H COSY correlations determined the partial structures of **1** including two distinct spin systems from H-3 (*δ*_H_ 7.13) to H_3_-8 (*δ*_H_ 1.14), and from H-9 (*δ*_H_ 5.98) to H_3_-11 (*δ*_H_ 1.54). Furthermore, the linkages between the partial structures were identified on the basis of the HMBC spectrum. The HMBC correlations of H-1 (*δ*_H_ 9.46), H-4 (*δ*_H_ 6.66), and H-10 (*δ*_H_ 5.92) to C-2 (*δ*_C_ 139.1), and those of H-3, H-9 to C-1 (*δ*_C_ 196.0) indicated that three parts of the compound were assembled via C-C bonds from C-2 to C-1, C-3, and C-9. Thus, the planar structure of **1** was determined as shown in Figure 1. The geometries of the Δ^2,4,9^-double bonds could be interpreted as 2*E*, 4*E*, and 9*Z* based on their ^3^*J*_H,H_ coupling constants of H-4/H-5 (*J* = 15.3 Hz) and H-9/H-10 (*J* = 11.8 Hz), as well as the strong NOESY correlations of H-3/H-1, H-3/H-5, and H-4/H_3_-11 (Figure 2).

**Figure 3:**
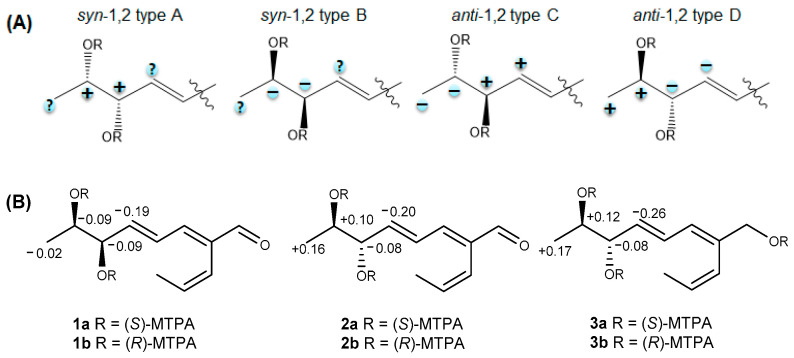
(**A**) Sign distribution (Δδ_S_ − _R_) of bis-MTPA esters of the four possible stereoisomers of 1,2-diols [16]; (**B**) Δδ_S_ − _R_ values of **1a**–**3b** in CD3OD.

The absolute configurations of four possible stereoisomers of the secondary 1,2-diols could be unambiguously determined using the modified Mosher’s method (Figure 3A) by comparing the ^1^H NMR data (*∆δ*_S-R_) of their corresponding bis-(*S*)- and -(*R*)-MTPA (α-methoxy-α-trifluoromethylphenylacetic acid) esters (Figure 3B) [16,17]. Therefore, compound **1** was treated with *R*- and *S*-*α*-methoxy-*α*-(trifluoromethyl) phenylacetyl chloride (MTPA-Cl) to give bis-*S*- and -*R*-MTPA esters (**1a** and **1b**), respectively. As a result, the Δ*δ_S−R_* values of **1a** and **1b** were interpreted, which were consistent with *syn*-1,2-diols (Figure 3A) and the absolute configurations of the stereogenic centers were determined as 6*R* and 7*R*. Thus, the structure of **1** was determined as (2*E*,4*E*,6*R*,7*R*)-6,7-dihydroxy-2-((*Z*)-prop-1-en-1-yl)octa-2,4-dienal and named talacyanol A.

Compound **2** was isolated as a colorless oil with the same molecular formula of C_11_H_16_O_3_ to that of **1**, which was established from an HRESIMS ion peak at *m*/*z* 219.1003 [M + Na]^+^ (calcd. for C_11_H_16_O_3_Na^+^, 219.0992). The one-dimensionial (1D) and two-dimensional (2D) NMR data of **2** were almost identical to those of **1**. By detailed analysis of NMR data, the planar structure of **2** was determined to be the same as that of **1** including the geometries of the double bounds (Figure 1). The only difference between **1** and **2** was the absolute stereochemistry of the secondary 1,2-diols at C-6 and C-7 according to their ^1^H NMR data in CDCl_3_ (*δ*_H-6_ 4.28 and *δ*_H-7_ 3.96 for **1**; *δ*_H-6_ 4.04 and *δ*_H-7_ 3.72 for **2**, Figure S23) and optical rotation values ${\left[\alpha \right]}_{D}^{25}$ +76.6 (*c* 0.2, MeOH) for **1**; ${\left[\alpha \right]}_{D}^{25}\;$−15 (*c* 0.2, MeOH) for **2**). By comparing ^1^H NMR data of bis-*S*- and -*R*-MTPA esters of **2**, the absolute configurations at C-6 and C-7 were assigned as 6*S* and 7*R* (Figure 3B), and the structure of **2** was determined as (2*E*,4*E*,6*S*,7*R*)-6,7-dihydroxy-2-((*Z*)-prop-1-en-1-yl)octa-2,4-dienal and named talacyanol B.

Compound **3** was obtained as a colorless oil with a molecular formula of C_11_H_18_O_3_ based on its HRESIMS data *m*/*z* 221.1155 [M + Na]^+^ (calcd for 221.1148, C_11_H_18_O_3_Na^+^). The ^1^H NMR spectrum of **3** was quite similar to that of **2**, the only difference lies in the chemical shift of the singlet proton H-1 (*δ*_H_ 9.46 in **2**, *δ*_H_ 4.05 in **3**), indicating that the aldehyde group at C-1 in **2** was replaced by a hydroxy group in **3**. Thus, the planar structure of **3** was elucidated as shown in Figure 1 based on its 1D and 2D NMR data.

A literature search revealed that the planar structure of **3** was similar to pinophol A, which was isolated from a plant endophytic fungus *Talaromyces pinophilus* by Zhao et al. [18]. However, the absolute configurations at C-6 and C-7 of pinophol A had not been determined yet and their relative configurations were reported as *syn*-1,2-diols (6*R*, 7*R* or 6*S*, 7*S*). Comparison of their optical rotation values (${\left[\alpha \right]}_{D}^{25}$ − 10 (*c* 0.2, MeOH) for **3** and ${\left[\alpha \right]}_{D}^{26}\;$+32.6 (*c* 0.1, MeOH) for pinophol A) and ^1^H NMR data in CDCl_3_ of **3** and pinophol A (*δ*_H-6_ 4.12 and *δ*_H-7_ 3.88 for **3** and *δ*_H-6_ 3.87 and *δ*_H-7_ 3.63 for pinophol A, Figure S22) suggested that they could be a pair of diastereomers. By analyzing ^1^H NMR data of tri-*S*- and -*R*-MTPA esters of **3**, the absolute configurations of C-6 and C-7 were determined as 6*S* and 7*R* (Figure 3B). Thus, the structure of **3** was determined as a new derivative of pinophol A, (2*E*,4*E*,6*S*,7*R*)-2-((*Z*)-prop-1-en-1-yl)octa-2,4-diene-1,6,7-triol, and named talacyanol C.

Aliphatic aldehydes are easily reduced to the corresponding alcohols in high yield by heterogeneous catalytic hydrogenation. To verify whether compound **3** is a true natural product or an artifact arising from compound **2** by reduction of aldehyde during the extraction process with ethyl acetate, we cultured the strain again and the culture broth was extracted successively with dichloromethane and n-butanol. Compound **3** was found in the butanol extract with a detectable concentration (Figures S31 and S32). Therefore, it could be concluded that **3** is a true natural substance.

The structures of the known compounds were identified as ramulosin (**4**), and eurothiocin A (**5**) by comparison of their spectroscopic data with those reported in the literature [19,20,21].

### 2.2. Bioactivities

Over the past few decades, emerging evidence has shown that many marine natural products, such as cytarabine, eribulin mesylate, brentuximab vedotin, and trabectidine exhibit beneficial effects in the prevention and treatment of cancer [22]. Furthermore, acyclic polyene polyols are a wide group of polyketides, and many of them display cytotoxicity against various cancer cell lines [17,23]. Therefore, compounds **1**–**5** were screened for in vitro cytotoxicity against six different cancer cell lines (stomach NUGC-3, colon HCT-15, lung NCI-H23, breast MDA-MB-231, prostate PC-3, and renal ACHN), the most common cancers in Korea [24]. Notably, compound **1** displayed moderate cytotoxicity against all the cancer cell lines with GI_50_ values ranging from 44.4 to 91.8 µM (Table 2). 

**Figure 4:**
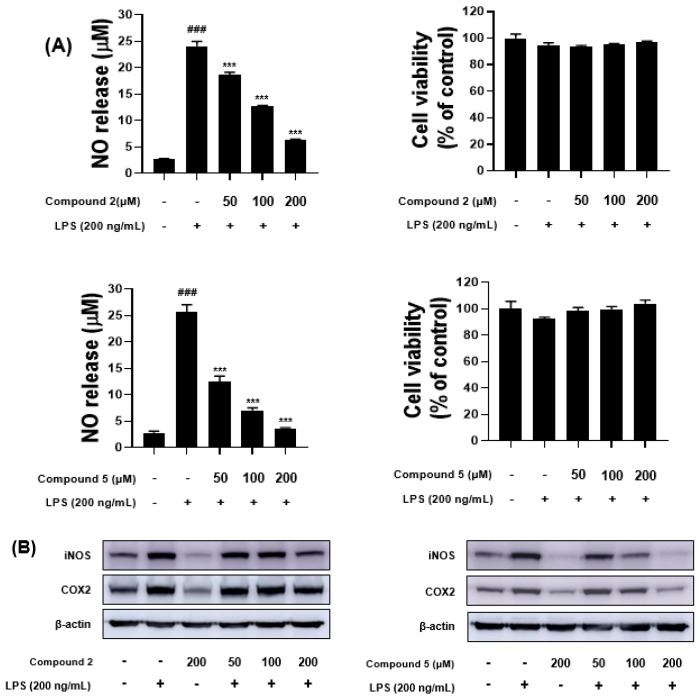
(**A**) The measurements of nitrite levels in the culture media were conducted using the Griess reaction. The release of NO was measured with indomethacin as a positive control (Figure S30). Cell viability was tested using the MTT (3-(4,5-dimetylthiazol-2-yl)-2,5-diphenyltetrazol bromide) assay. Results are shown as the percentage of control samples; (**B**) Inhibition of inducible nitric oxide synthase (iNOS) and cyclooxygenase-2 (COX-2) protein and mRNA expression by compounds **2** and **5** in lipopolysaccharide (LPS)-stimulated BV-2 cells. The data (B) is expressed as the relative signal intensity for two independent experiments. Values are the mean ± standard error. ^###^ *p* < 0.001, vs. control group and *** *p* < 0.001 vs. LPS-treated group.

Compounds **1**–**5** were also tested for their inhibitory effects on the production of nitric oxide (NO) in lipopolysaccharide (LPS)-stimulated BV-2 microglial cells. The cells were initially treated with a high concentration (200 µM) of each compound and LPS (200 ng/mL) to screen their inhibitory effect on NO production. All the compounds showed weak or strong inhibitory effects on NO production (Figure S33), and compounds **2** and **5** showed the most potent anti-inflammatory activity. Therefore, talacyanol B (**2**) and eurothiocin A (**5**) were selected for further studies to investigate NO production in BV-2 cells and LPS-induced expression levels of cyclooxygenase-2 (COX-2) and inducible nitric oxide synthase (iNOS) proteins by Western blot analysis. As shown in Figure 4A,B, the NO production and expression levels of COX-2 and iNOS proteins were suppressed by both **2** and **5** in a dose-related fashion at the concentrations of 50, 100, and 200 µM.

## 3. Experimental Methods

### 3.1. General Experimental Procedures

The 1D (^1^H and ^13^C) and 2D (COSY, HSQC, HMBC, and NOESY) NMR spectra were acquired by a Bruker 600 MHz spectrometer (Bruker BioSpin GmbH, Rheinstetten, Germany). Specific optical rotations were obtained in methanol at 25 °C on a Rudolph Research Analytical (Autopol III) polarimeter (Rudolph Research Analytical, Hackettstown, NJ, USA). UV-visible spectra were acquired by a Shimadzu UV-1650PC spectrophotometer in 1 mm quartz cells (Shimadzu Corporation, Kyoto, Japan). IR spectra were collected on a JASCO FT/IR-4100 spectrophotometer (JASCO Corporation, Tokyo, Japan). High-resolution ESIMS were recorded on a hybrid ion-trap time-of-flight mass spectrometer (Shimadzu LC/MS-IT-TOF). HPLC was conducted using a semi-prep ODS column (YMC-Triart C_18_, 250 × 10 mm i.d, 5 µm) and an analytical ODS column (YMC-Triart C_18_, 250 × 4.6 mm i.d, 5 µm) (YMC Corporation, Kyoto, Japan). All the reagents were purchased from Sigma-Aldrich (Merck KGaA, Darmstadt, Germany), and the organic solvents and water were distilled prior to use.

### 3.2. Fungal Material and Fermentation

The fungal strain 168ST-51.1 was isolated from the seaweed *Caulerpa* sp. collected in Son Tra peninsular, Da Nang, Vietnam in August 2016. The fungus was identified as *Talaromyces cyanescens* Stchigel & Guarro on the basis of DNA amplification and ITS gene sequencing (GenBank accession number MK 072976.1). The voucher of this strain is currently deposited in the Microbial Culture Collection, KIOST, with the name of *Talaromyces* sp. 168ST-51.1 under the curatorship of Hee Jae Shin.

The seed and mass cultures were performed in Bennett’s medium (1% glucose, 0.2% tryptone, 0.1% yeast extract, 0.1% beef extract, 0.5% glycerol, sea salts 32 g/L, and agar 17 g/L for agar medium). The fungus was initially cultured on Bennett’s agar medium in a Petri dish for 7 days. The actively grown mycelium was transferred aseptically into a 500 mL conical flask containing 300 mL of Bennett’s broth medium and incubated on a rotary shaker (140 rpm) at 28 °C for 4 days. An aliquot (0.1% *v*/*v*) from the seed culture was inoculated into twenty 2 L flasks each containing 1 L of the medium and grown under the same conditions as described for the seed culture for 7 days, and then harvested.

### 3.3. Extraction and Isolation of Metabolites

After cultivation, the culture broth was extracted with ethyl acetate (20 L × 2 times). The organic layer was evaporated under vacuum at 37 °C to yield a crude extract (3.0 g). Afterwards, the crude extract was separated into fifteen fractions (Fr. 1 to Fr. 15) by vacuum liquid chromatography on a flash ODS column (20 cm × 4.5 cm), which was stepwise eluted with 3 × 250 mL each of 20%, 40%, 60%, 80% MeOH in H_2_O, and 100% MeOH. Fraction 4 was purified by an analytical RP-HPLC (YMC-Pack-ODS-A, 250 × 4.6 mm i.d, 5 µm) using an isocratic condition with 14% ACN in H_2_O at a flow rate of 1 mL/min to afford compound **3** (3.0 mg, *t*_R_ = 15 min). Fraction 5 was applied to a semi-preparative RP-HPLC (YMC-Pack-ODS-A, 250 × 10 mm i.d, 5 µm, flow rate 2.0 mL/min) using an isocratic elution with 14% ACN in H_2_O to yield compounds **2** (20.0 mg, *t*_R_ = 38 min) and **1** (5.0 mg, *t*_R_ = 42 min). Fraction 9 was recrystallized from methanol to give compound **4** (10.0 mg) as yellow needles. Finally, compound **5** (4.0 mg) was purified from fraction 10 using a semi-preparative RP-HPLC (YMC-Pack-ODS-A, 250 × 10 mm i.d, 5 µm, flow rate 2.0 mL/min) with an isocratic elution of 37% ACN in H_2_O for 46 min. All the purification procedure was repeated 2 times to yield sufficient amounts of **1**–**5** for structure determination and bioassays.

#### 3.3.1. Talacyanol A (**1**)

Colorless oil, ${\left[\alpha \right]}_{D}^{25}$ +76.6 (*c* 0.2, MeOH). UV (MeOH) λ_max_ (log ε) 285 (2.86), 232 (2.38) nm; IR (MeOH) ν_max_ 3392 (br), 2975, 2933, 1671, 1632 cm^−1^; HRESIMS *m*/*z* 219.1002 [M + Na]^+^ (calcd for 219.0992, C_11_H_16_O_3_Na^+^); ^1^H NMR (CD_3_OD, 600 MHz) and ^13^C NMR (CD_3_OD, 150 MHz) see Table 1.

#### 3.3.2. Talacyanol B (**2**)

Colorless oil, ${\left[\alpha \right]}_{D}^{25}$ −15 (*c* 0.2, MeOH). UV (MeOH) λ_max_ (log ε) 285 (2.86), 232 (2.38) nm; IR (MeOH) ν_max_ 3392 (br), 2975, 2939, 1671, 1632 cm^−1^; HRESIMS *m*/*z* 219.1003 [M + Na]^+^ (calcd for 219.0992, C_11_H_16_O_3_Na^+^); ^1^H NMR (CD_3_OD, 600 MHz) and ^13^C NMR (CD_3_OD, 150 MHz) see Table 1.

#### 3.3.3. Talacyanol C (**3**)

Colorless oil, ${\left[\alpha \right]}_{D}^{25}$ −10 (*c* 0.2, MeOH). UV (MeOH) λ_max_ (log ε) 248 (2.61), 204 (1.56) nm; IR (MeOH) ν_max_ 3335 (br), 2971, 2943, 1056 cm^−1^; HRESIMS *m*/*z* 221.1155 [M + Na]^+^ (calcd for 221.1148, C_11_H_18_O_3_Na^+^); ^1^H NMR (CD_3_OD, 600 MHz) and ^13^C NMR (CD_3_OD, 150 MHz) see Table 1.

### 3.4. MTPA Esterification of Compounds ***1**–**3***

Compound **1** (1.0 mg for each) was dissolved in anhydrous pyridine (200 µL), and then added dimethylaminopyridine (DMAP). Afterwards, (*R*)-MTPA-Cl (20 μL) or (*S*)-MTPA-Cl (20 μL) were introduced, and reaction mixture was stirred at ambient temperature for 30 min, and then quenched with MeOH. Each mixture ((a) **1** with **1a** and (b) **1** with **1b**) was dried to dryness and purified by analytical reversed-phase HPLC to afford **1a** and **1b**. (*R*)- and (*S*)-MTPA esters of compounds **2** and **3** were prepared in the same procedure as described above for compound **1.** The Δ*δ_S-R_* values around the stereogenic centers of the MTPA esters were determined by ^1^H, HSQC, and ^1^H-^1^H COSY NMR spectra.

#### 3.4.1. Bis-*S*-MTPA Ester (**1a**) of Talacyanol A (**1**)

^1^H NMR (600 MHz, CD_3_OD) *δ* 9.47 (s, 1H), 7.39–7.51 (m, 10H), 6.87 (d, *J* = 11.1, 1H), 6.54 (dd, *J* = 11.7, 14.9, 1H), 6.04 (dd, *J* = 6.3, 15.4, 1H), 5.86 (m, 2H), 5.74 (dd, *J* = 2.8, 6.3, 1H), 5.36 (m, 1H), 3.46 (s, 3H), 3.45 (s, 3H), 1.40 (d, *J* = 5.1, 3H), 1.28 (d, *J* = 6.5, 3H).

#### 3.4.2. Bis-*R*-MTPA Ester (**1b**) of Talacyanol A (**1**)

^1^H NMR (600 MHz, CD_3_OD) *δ* 9.47 (s, 1H), 7.39–7.49 (m, 10H), 7.02 (d, *J* = 11.2, 1H), 6.52 (dd, *J* = 10.9, 15.1, 1H), 6.23 (dd, *J* = 5.9, 15.5, 1H), 5.84 (m, 2H), 5.83 (m, 1H), 5.45 (m, 1H), 3.44 (s, 3H), 3.43 (s, 3H), 1.40 (d, *J* = 5.4, 3H), 1.30 (d, *J* = 6.6, 3H).

#### 3.4.3. Bis-*S*-MTPA Ester (**2a**) of Talacyanol B (**2**)

^1^H NMR (600 MHz, CD_3_OD) *δ* 9.47 (s, 1H), 7.32–7.49 (m, 10H), 6.98 (d, *J* = 11.2, 1H), 6.51 (dd, *J* = 11.0, 15.4, 1H), 6.12 (dd, *J* = 6.9, 15.4, 1H), 5.88 (m, 2H), 5.82 (dd, *J* = 2.5, 5.9, 1H), 5.52 (m, 1H), 3.47 (s, 3H), 3.44 (s, 3H), 1.47 (d, *J* = 5.2, 3H), 1.35 (d, *J* = 6.6, 3H).

#### 3.4.4. Bis-*R*-MTPA Ester (**2b**) of Talacyanol B (**2**)

^1^H NMR (600 MHz, CD_3_OD) *δ* 9.49 (s, 1H), 7.33–7.48 (m, 10H), 7.08 (d, *J* = 11.1, 1H), 6.73 (dd, *J* = 11.0, 15.4, 1H), 6.32 (dd, *J* = 7.0, 15.5, 1H), 5.94 (m, 2H), 5.90 (dd, *J* = 2.7, 6.8, 1H), 5.42 (m, 1H), 3.43 (s, 3H), 3.41 (s, 3H), 1.47 (d, *J* = 5.1, 3H), 1.19 (d, *J* = 6.6, 3H).

#### 3.4.5. Tri-*S*-MTPA Ester (**3a**) of Talacyanol C (**3**)

^1^H NMR (600 MHz, CD_3_OD) 7.28–7.51 (m, 15H), 6.33 (dd, *J* = 10.8, 15.3, 1H), 6.08 (d, *J* = 10.9, 1H), 5.77 (m, 2H), 5.65 (dd, *J* = 2.2, 7.6, 1H), 5.47 (dd, *J* = 7.58, 15.3, 1H), 5.44 (m, 1H), 4.88 (d, *J* = 10.3, 1H), 4.82 (d, *J* = 14.0, 1H), 3.52 (s, 3H), 3.44 (s, 3H), 3.44 (s, 3H), 1.48 (d, *J* = 5.3, 3H), 1.31 (d, *J* = 6.6, 3H).

#### 3.4.6. Tri-*R*-MTPA Ester (**3b**) of Talacyanol C (**3**)

^1^H NMR (600 MHz, CD_3_OD) 7.32–7.50 (m, 15H), 6.47 (m, 1H), 6.15 (d, *J* = 10.9, 1H), 6.04 (dd, *J* = 6.3, 15.4, 1H), 5.80 (m, 2H), 5.73 (m, 2H), 5.34 (m, 1H), 4.91 (d, *J* = 12.8, 1H), 4.80 (d, *J* = 12.8, 1H), 3.51 (s, 3H), 3.42 (s, 3H), 3.39 (s, 3H), 1.44 (d, *J* = 5.4, 3H), 1.14 (d, *J* = 6.5, 3H).

### 3.5. Cytotoxicity Test by SRB Assay and Anti-Neuroinflammatory Test

The SRB cytotoxicity assay and anti-neuroinflammatory test for compounds **1**–**5** were performed as previously described [25,26].

## 4. Conclusions

Chemical examination of the ethyl acetate extract of the marine-derived fungus *Talaromyces cyanescens* 168ST-51.1 led to the isolation of three new compounds, talacyanol A–C (**1**–**3**), together with two known compounds **4** and **5**. The structures of the new compounds **1**–**3** were determined by the spectroscopic and modified Mosher’s methods. The known compounds **4**–**5** were identified by comparing their spectroscopic data with those reported in literature. Talacyanol A (**1**) expressed *in vitro* cytotoxicity against various cancer cell lines. In contrast, talacyanol B (**2**) and eurothiocin A (**5**) showed anti-neuroinflammatory activity without cytotoxicity. The results demonstrated that the absolute configurations of the chiral centers may exert significant effects on biological activities of natural products. To the best of our knowledge, this is the first report on the cytotoxicity of compound **1** and the anti-neuroinflammatory effect of compounds **2** and **5**.

## Figures and Tables

**Table 1:** ^1^H and ^13^C NMR data for **1**–**3** at 600 MHz and 150 MHz in CD_3_OD (δ in ppm, *J* in Hz), respectively.

Position	1		2		3	
	*δ*_H_ (*J* in Hz)	*δ*_C_	*δ*_H_ (*J* in Hz)	*δ*_C_	*δ*_H_ (*J* in Hz)	*δ*_C_
1	9.46, s	196.0	9.46, s	196.0	4.05, s	66.4
2		139.1		139.1		139.8
3	7.13 (d, 11.2)	150.2	7.13 (d, 11.2)	150.3	6.20 (d, 11.0)	126.6
4	6.66 (dd, 11.3, 15.3)	128.8	6.63 (dd, 11.2, 15.3)	128.6	6.33 (dd, 11.0, 15.3)	130.7
5	6.44 (dd, 5.6, 15.3)	146.0	6.49 (dd, 5.6, 15.3)	146.3	5.80 (dd, 7.0, 15.4)	134.1
6	4.09 (t, 5.5)	77.0	4.07 (t, 5.4)	77.0	3.93 (t, 5.5)	77.7
7	3.72, m	71.4	3.71, m	71.5	3.67, m	71.7
8	1.14 (d, 6.4)	18.7	1.17 (dd, 1.0, 6.4)	18.9	1.13 (d, 6.4)	18.6
9	5.98 (d, 11.8)	122.0	5.98 (d, 11.8)	122.0	5.90 (d, 11.5)	127.1
10	5.92, m	132.5	5.92, m	132.4	5.75, m	129.9
11	1.54 (d, 6.6)	15.7	1.54 (d, 6.6)	15.7	1.61 (dd, 1.8, 6.9)	15.4

**Table 2:** Growth inhibition (GI_50_, μM) values of **1** against human tumor cell lines.

Cell Line	GI_50_, μM	ADR ^a^
HCT-15	64.3	<0.5
NUGC-3	62.2	<0.5
NCI-H23	70.9	<0.5
ACHN	44.4	<0.5
PC-3	54.1	<0.5
MDA-MB-231	91.8	<0.5

GI_50_ values are the concentration corresponding to 50% growth inhibition. ^a^ ADR, adriamycin as standard.

## Data Availability

The Data presented in the article are available in the supplementary materials.

## Supplementary Materials

The following materials are available online, Figures S1–S23: HRESI-MS data, ^1^H NMR, ^13^C NMR, ^1^H-^1^H COSY, HSQC, HMBC, NOESY experimental spectra of compounds **1**–**3**, Figures S24–S29: ^1^H NMR spectra of *R-* and *S*-MTPA esters of compounds **1**–**3**.

## Sample Availability

Samples of the compounds are available from the authors.
