# Supplementary material for: New Polyenes from the Marine-Derived Fungus Talaromyces cyanescens with Anti-Neuroinflammatory and Cytotoxic Activities
Source: Molecules. 2021 Feb 5;26(4):836. doi: 10.3390/molecules26040836 (PMC7915668; doi:10.3390/molecules26040836)
Supplement: Supplementary file 1 [file molecules-26-00836-s001.pdf]

Supplementary data

New Polyenes from the Marine-derived Fungus *Talaromyces cyanescens* with Anti-Neuroinflammatory and Cytotoxic Activities

Hee Jae Shin [1,2,*], Cao Van Anh [1,2], Duk-Yeon Cho [3], Dong-Kug Choi [3], Jong Soon Kang [4], Phan Thi Hoai Trinh [5], Byeoung-Kyu Choi [1] and Hwa-Sun Lee [1]

[1] Marine Natural Products Chemistry Laboratory, Korea Institute of Ocean Science and Technology, 385 Haeyang-ro, Yeongdo-gu, Busan 49111, Korea; caovananh@kiost.ac.kr (C.V.A.); choibk4404@kiost.ac.kr (B.-K.C.); hwasunlee@kiost.ac.kr (H.-S.L.);

[2] Department of Marine Biotechnology, University of Science and Technology (UST), 217 Gajungro, Yuseong-gu, Daejeon 34113, Korea

[3] Department of Applied Life Science, Graduate school, BK21 Program, Konkuk University, Chungju 27478, Korea; whejrdus10@kku.ac.kr (D.-Y.C.); choidk@kku.ac.kr (D.-K.C.)

[4] Laboratory Animal Resource Center, Korea Research Institute of Bioscience and Biotechnology, 30 Yeongudanjiro, Cheongju 28116, Korea; kanjon@kribb.re.kr (J.S.K.)

[5] Department of Marine Biotechnology, Nhatrang Institute of Technology Research and Application, Vietnam Academy of Science and Technology, 02 Hung Vuong, Nha Trang 650000, Vietnam; phanhoaitrinh84@gmail.com (P.T.H.T.);

Contents

Figure S1. HR-ESIMS data of talacyanol A (**1**)……………………………………………………………3
Figure S2. ^{1}H NMR (600 MHz, CD_3OD) spectrum of talacyanol A (**1**)……………………………………4
Figure S3. ^{13}C NMR (150 MHz, CD_3OD) spectrum of talacyanol A (**1**)……………………………………4
Figure S4. COSY spectrum of talacyanol A (**1**)…………………………………………………………………5
Figure S5. HSQC spectrum of talacyanol A (**1**)…………………………………………………………………7
Figure S6. HMBC spectrum of talacyanol A (**1**). ………………………………………………………………8
Figure S7. NOESY spectrum of talacyanol A (**1**). ……………………………………………………………9
Figure S8. HR-ESIMS data of talacyanol B (**2**). ……………………………………………………………11
Figure S9. ^{1}H NMR (600 MHz, CD_3OD) spectrum of talacyanol B (**2**)……………………………………12
Figure S10. ^{13}C NMR (150 MHz, CD_3OD) spectrum of talacyanol B (**2**)…………………………………12
Figure S11. COSY spectrum of talacyanol B (**2**)………………………………………………………………13
Figure S12. HSQC spectrum of talacyanol B (**2**) ……………………………………………………………15
Figure S13. HMBC spectrum of talacyanol B (**2**)……………………………………………………………16
Figure S14. NOESY spectrum of talacyanol B (**2**). …………………………………………………………17
Figure S15. HR-ESIMS data of talacyanol C (**3**)……………………………………………………………19
Figure S16. ^{1}H NMR (600 MHz, CD_3OD) spectrum of talacyanol C (**3**)……………………………………20
Figure S17. ^{13}C NMR (150 MHz, CD_3OD) spectrum of talacyanol C (**3**)…………………………………20
Figure S18. COSY spectrum of talacyanol C (**3**)………………………………………………………………21
Figure S19. HSQC spectrum of talacyanol C (**3**)………………………………………………………………23
Figure S20. HMBC spectrum of talacyanol C (**3**)……………………………………………………………24
Figure S21. NOESY spectrum of talacyanol C (**3**). …………………………………………………………25
Figure S22. ^{1}H NMR (600 MHz, $CDCl_3$) spectrum of talacyanol C (**3**)……………………………………27
Figure S23. Comparison of ^{1}H NMR (600 MHz, $CDCl_3$) spectra between talacyanols A (**1**) and B (**2**)……………………………………………………………………………………………………27
Figure S24. ^{1}H NMR (600 MHz, CD_3OD) spectrum of bis-(*S*)-MTPA ester of **1** (**1a**)……………………28
Figure S25. ^{1}H NMR (600 MHz, CD_3OD) spectrum of bis-(*R*)-MTPA ester of **1** (**1b**)……………………29
Figure S26. ^{1}H NMR (600 MHz, CD_3OD) spectrum of bis-(*S*)-MTPA ester of **2** (**2a**)……………………30
Figure S27. ^{1}H NMR (600 MHz, CD_3OD) spectrum of bis-(*R*)-MTPA ester of **2** (**2b**)……………………31
Figure S28. ^{1}H NMR (600 MHz, CD_3OD) spectrum of tri-(*S*)-MTPA ester of **3** (**3a**)……………………32
Figure S29. ^{1}H NMR (600 MHz, CD_3OD) spectrum of tri-(*R*)-MTPA ester of **3** (**3b**)……………………33
Figure S30. NO release level with 25~200 μM of indomethacin as a positive control……………………34
Figure S31. Comparision of ^{1}H NMR (600 MHz, CD_3OD) spectra between ethyl acetate, dichloromethane, and butanol extracts containing compound **3**……………………………………35
Figure S32. Detection of talacyanol C (**3**) in the butanol extract by HPLC-MS-UV……………………36
Figure S33. The inhibitory effect on NO production of compounds **1-5** at a concentration of 200 μM....37

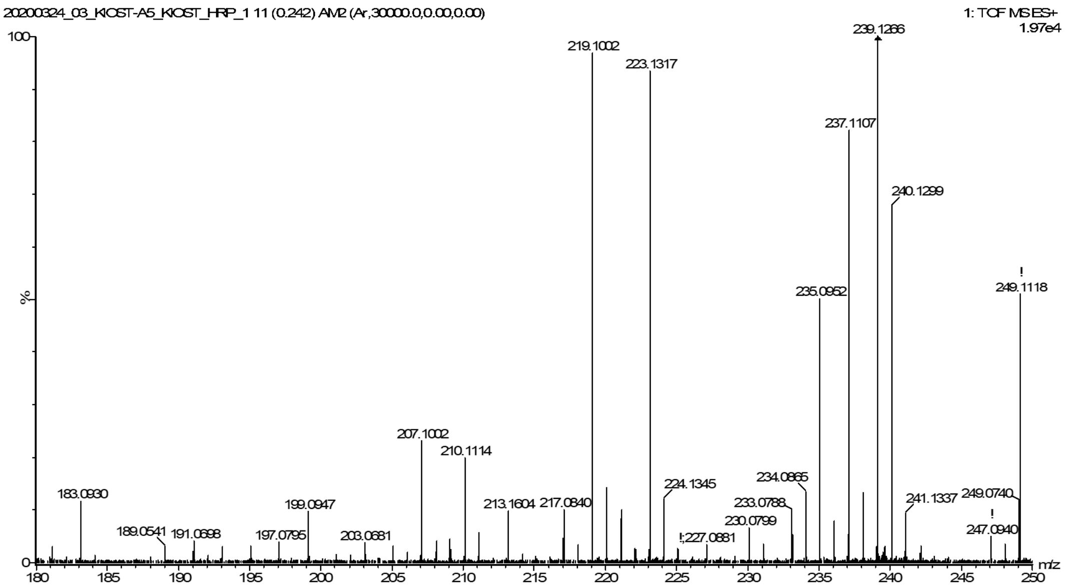

Monoisotopic Mass, Even Electron Ions
11 formula(e) evaluated with 1 results within limits (all results (up to 1000) for each mass)
Elements Used:
C: 0-15 H: 0-20 O: 0-3 Na: 0-1

Minimum:				-1.5				
Maximum:		500.0	5.0	100.0				
Mass	Calc. Mass	mDa	PPM	DBE	i-FIT	Norm	Conf(%)	Formula
219.1002	219.0992	0.5	2.3	3.5	673.2	n/a	n/a	C11 H16 O3 Na+

Figure S1. HR-ESIMS data of talacyanol A (**1**).

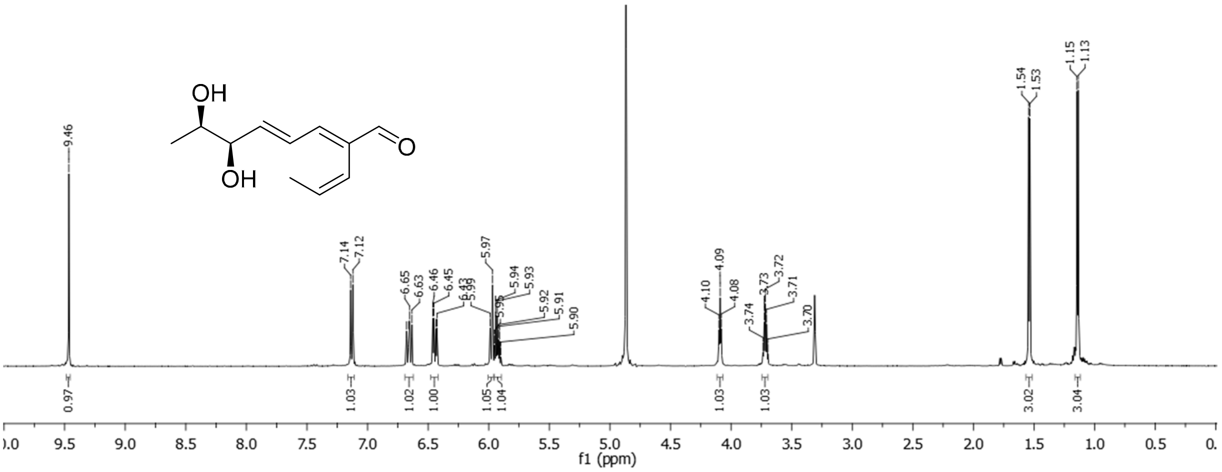

Figure S2. ^{1}H NMR (600 MHz, CD_3OD) spectrum of talacyanol A (**1**).

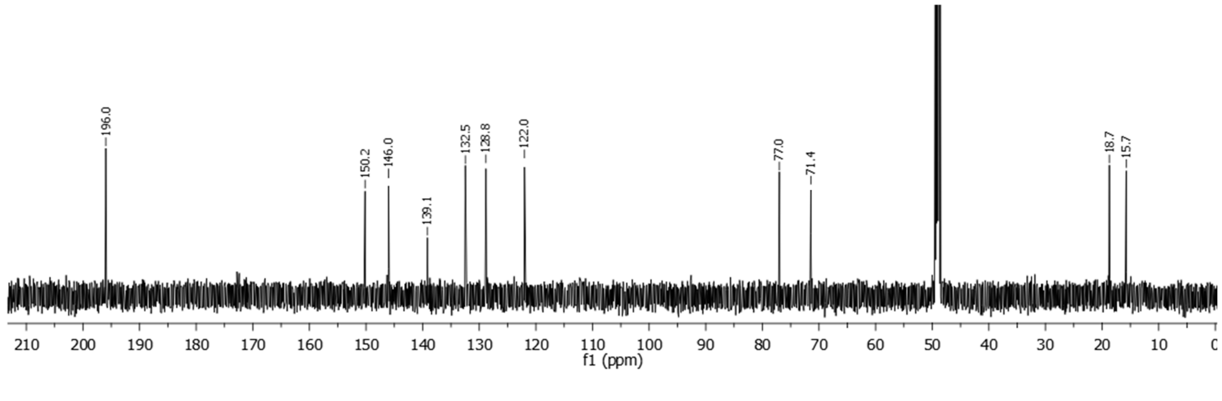

Figure S3. ^{13}C NMR (150 MHz, CD_3OD) spectrum of talacyanol A (**1**).

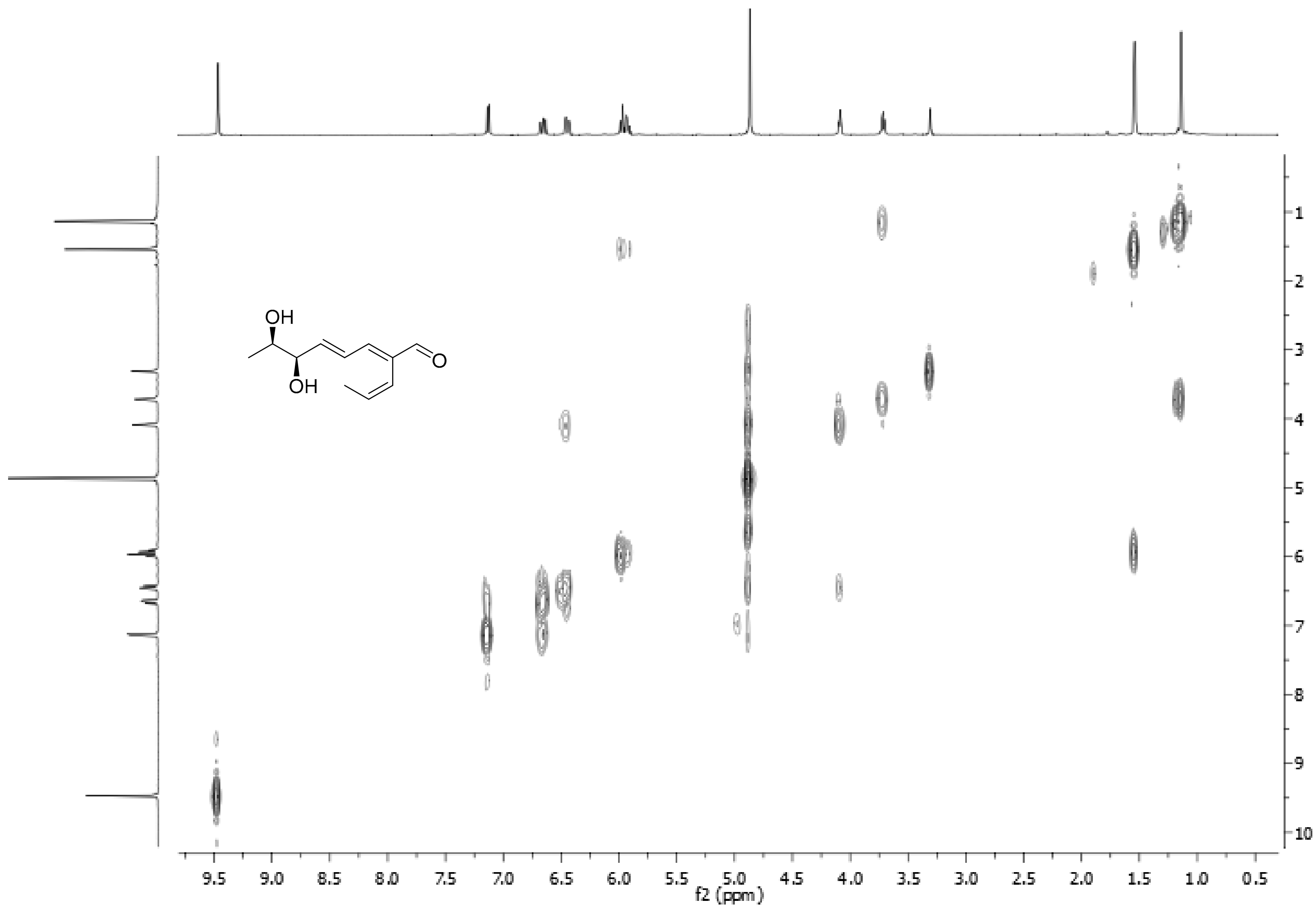

Figure S4. COSY spectrum of talacyanol A (**1**).

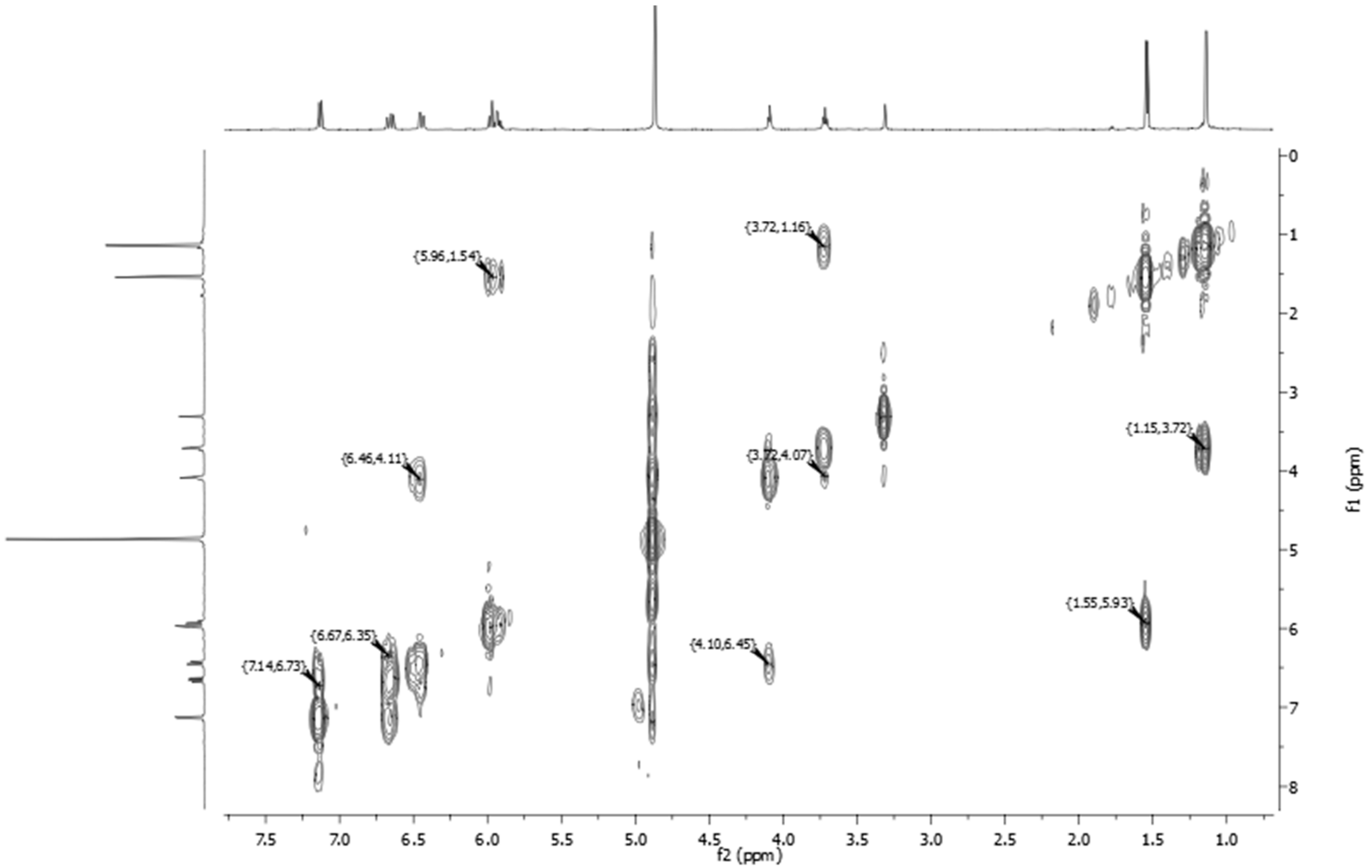

COSY spectrum of **1** (zoomed)

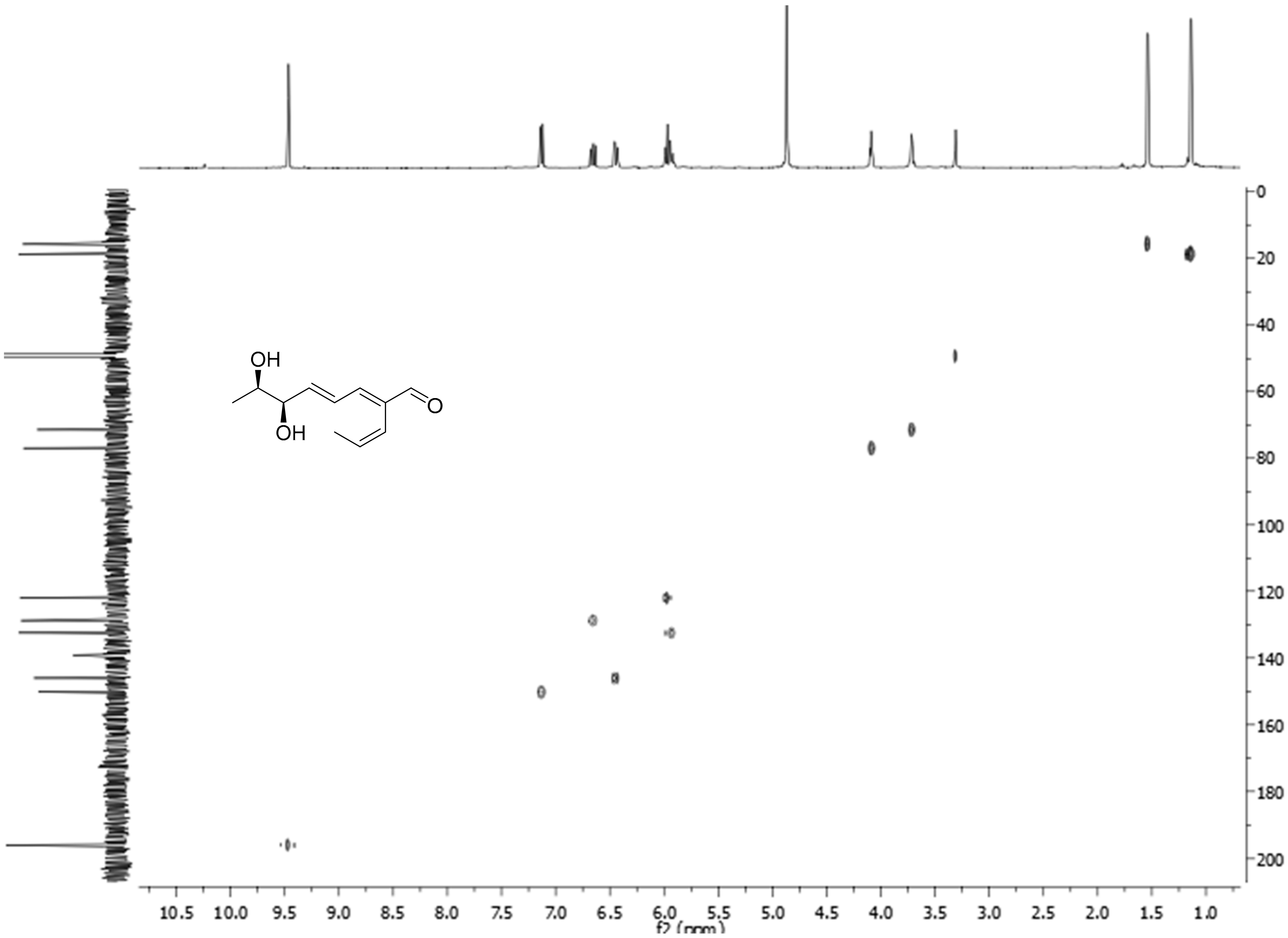

Figure S5. HSQC spectrum of talacyanol A (**1**).

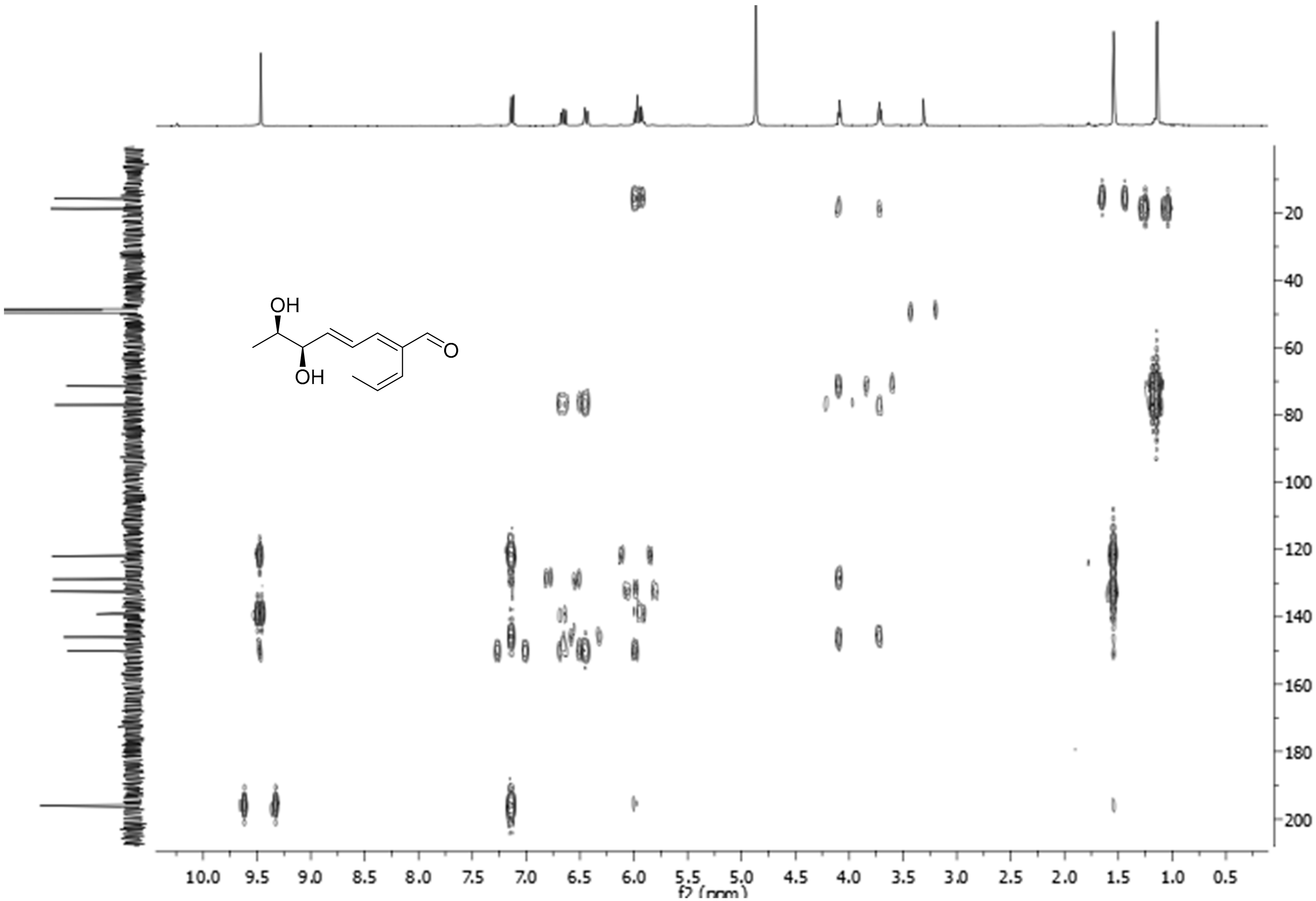

Figure S6. HMBC spectrum of talacyanol A (**1**).

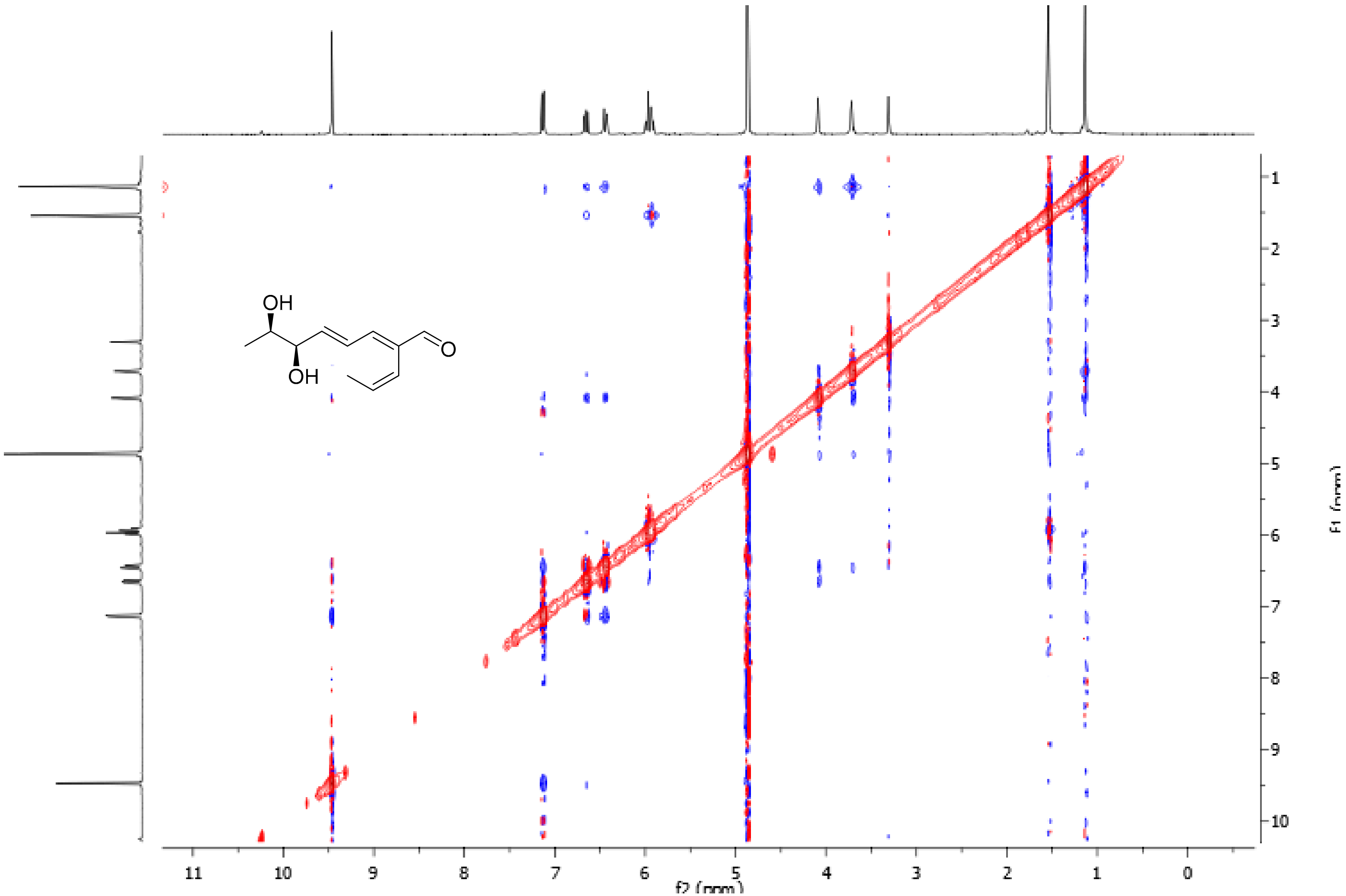

Figure S7. NOESY spectrum of talacyanol A (**1**).

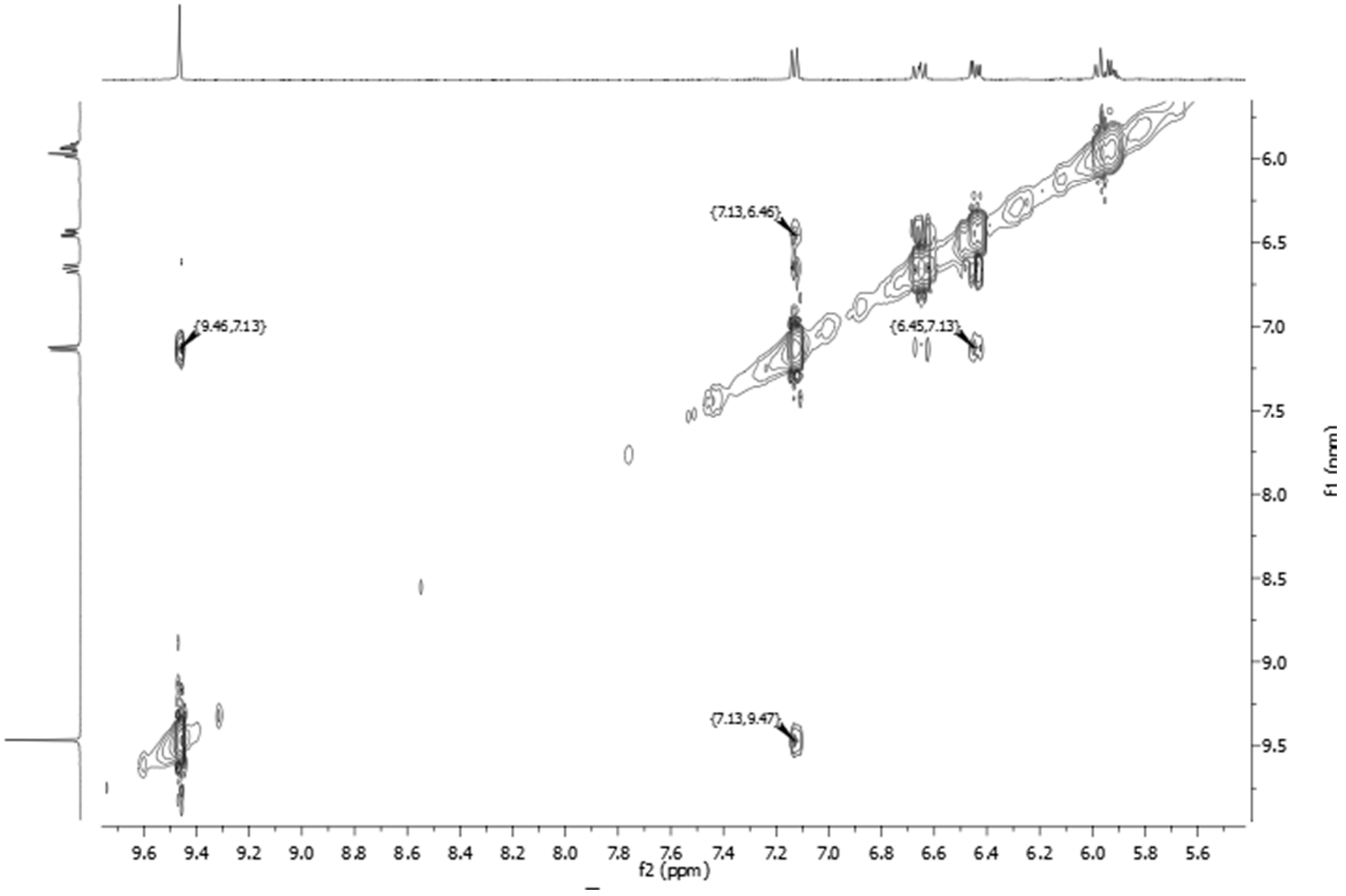

NOESY spectrum of **1** (zoomed)

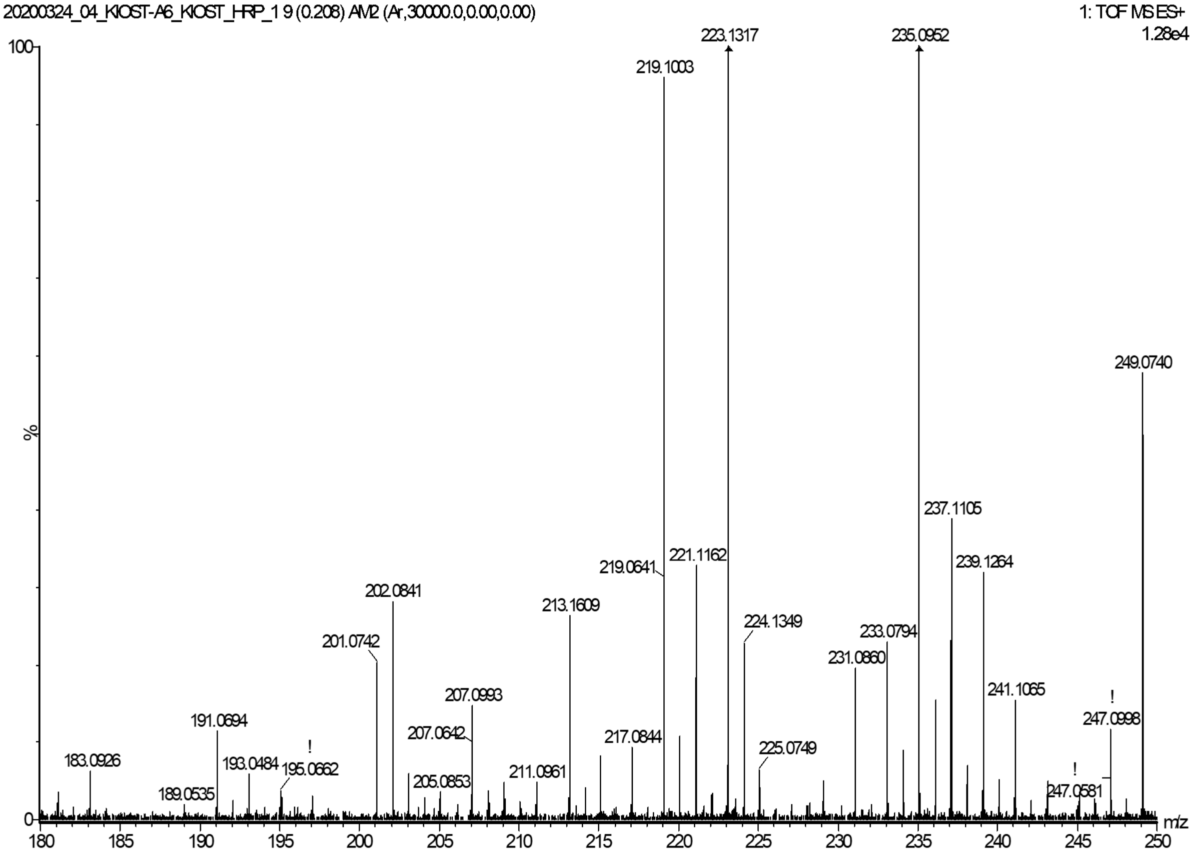

Monoisotopic Mass, Even Electron Ions
11 formula(e) evaluated with 1 results within limits (all results (up to 1000) for each mass)
Elements Used:
C: 0-15 H: 0-20 O: 0-3 Na: 0-1

Minimum:				-1.5				
Maximum:		500.0	5.0	100.0				
Mass	Calc. Mass	mDa	PPM	DBE	i-FIT	Norm	Conf(%)	Formula
219.1003	219.0992	0.6	2.7	3.5	635.3	n/a	n/a	$C_{11}H_{16}O_3Na^+$

Figure S8. HR-ESIMS data of talacyanol B (**2**).

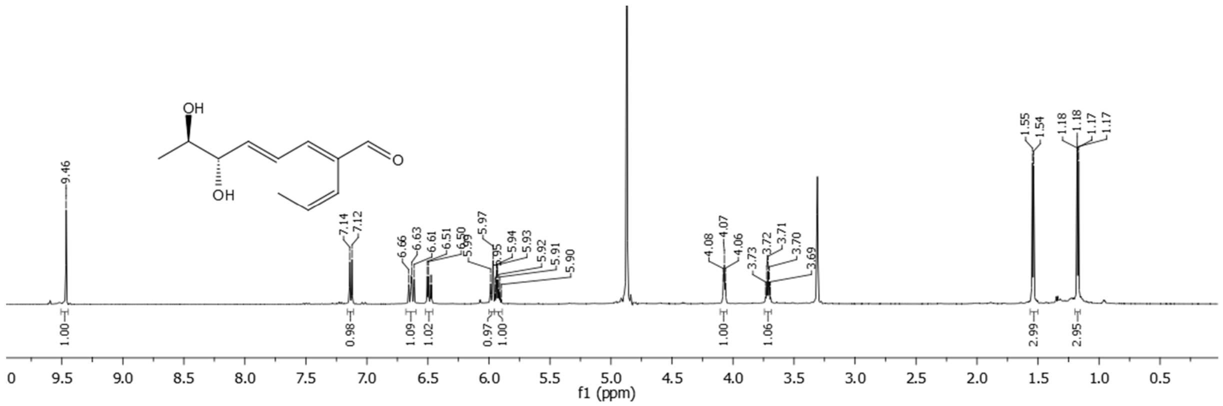

Figure S9. ^{1}H NMR (600 MHz, CD3OD) spectrum of talacyanol B (**2**).

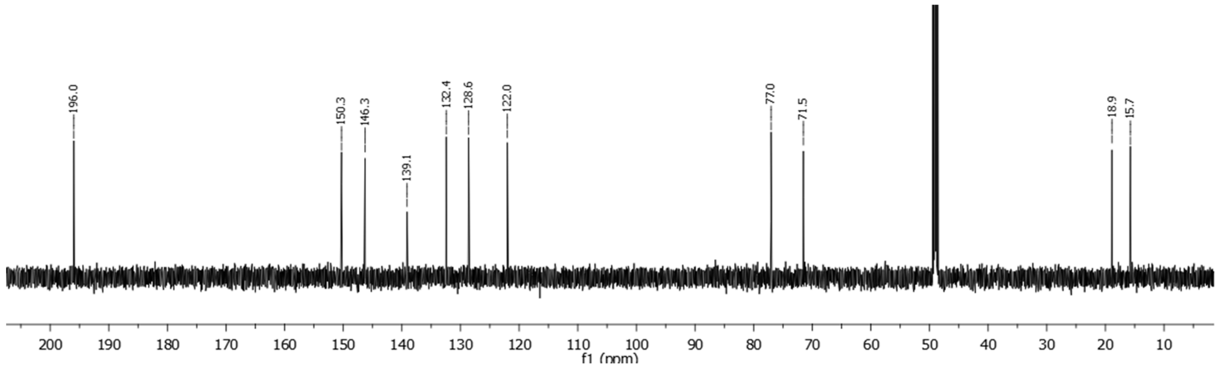

Figure S10. ^{13}C NMR (150 MHz, CD3OD) spectrum of talacyanol B (**2**).

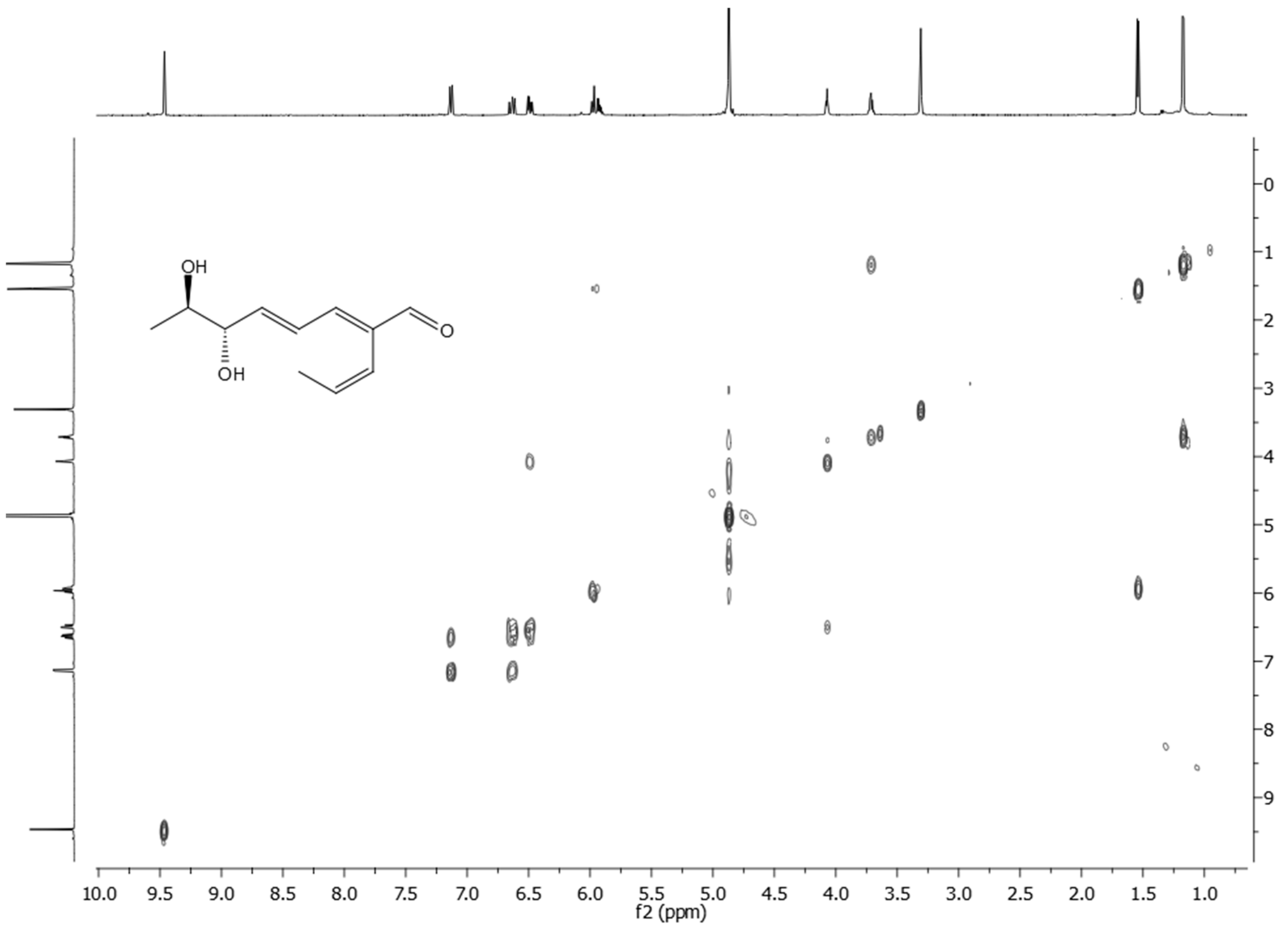

Figure S11. COSY spectrum of talacyanol B (**2**).

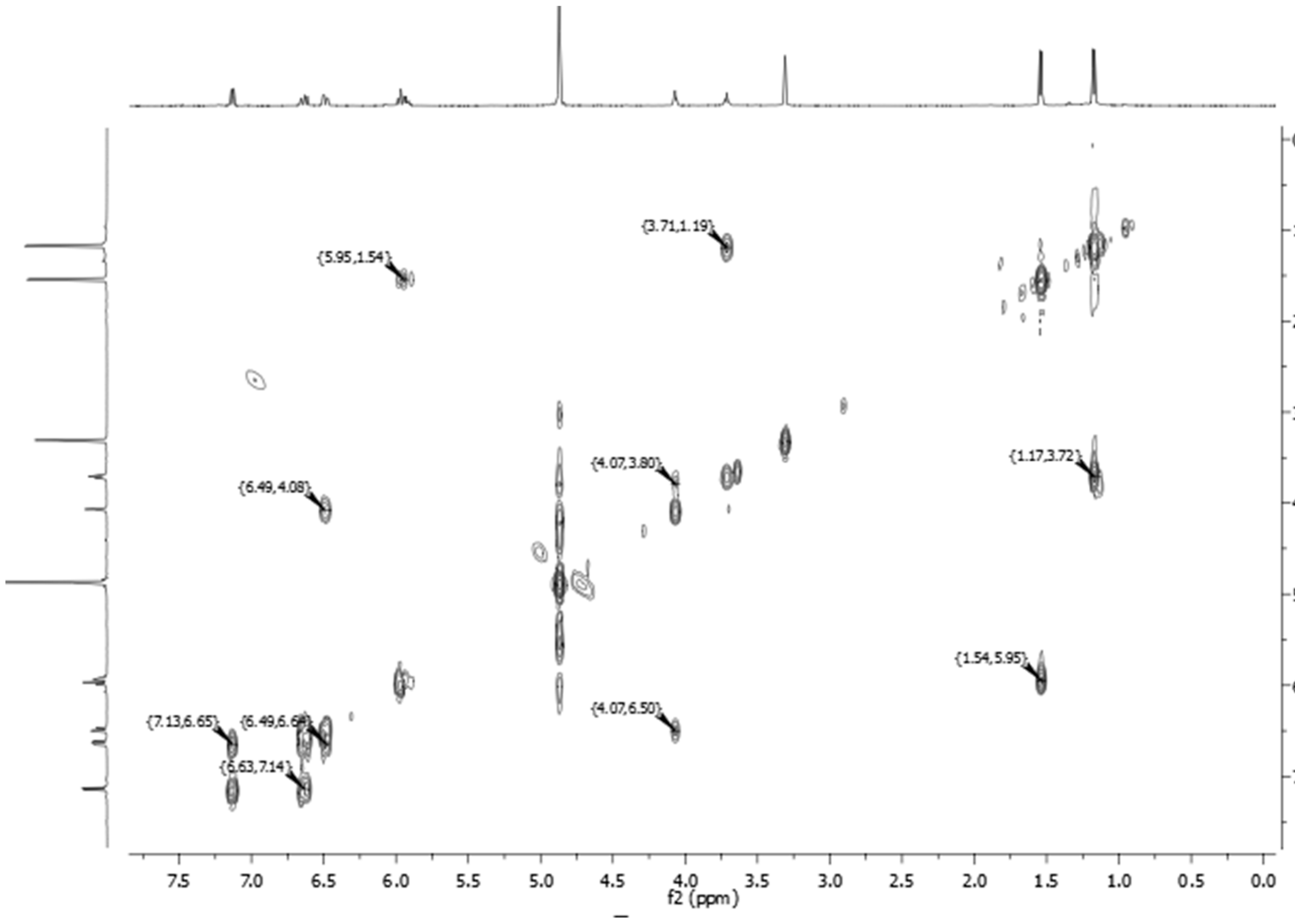

COSY spectrum of **2** (zoomed).

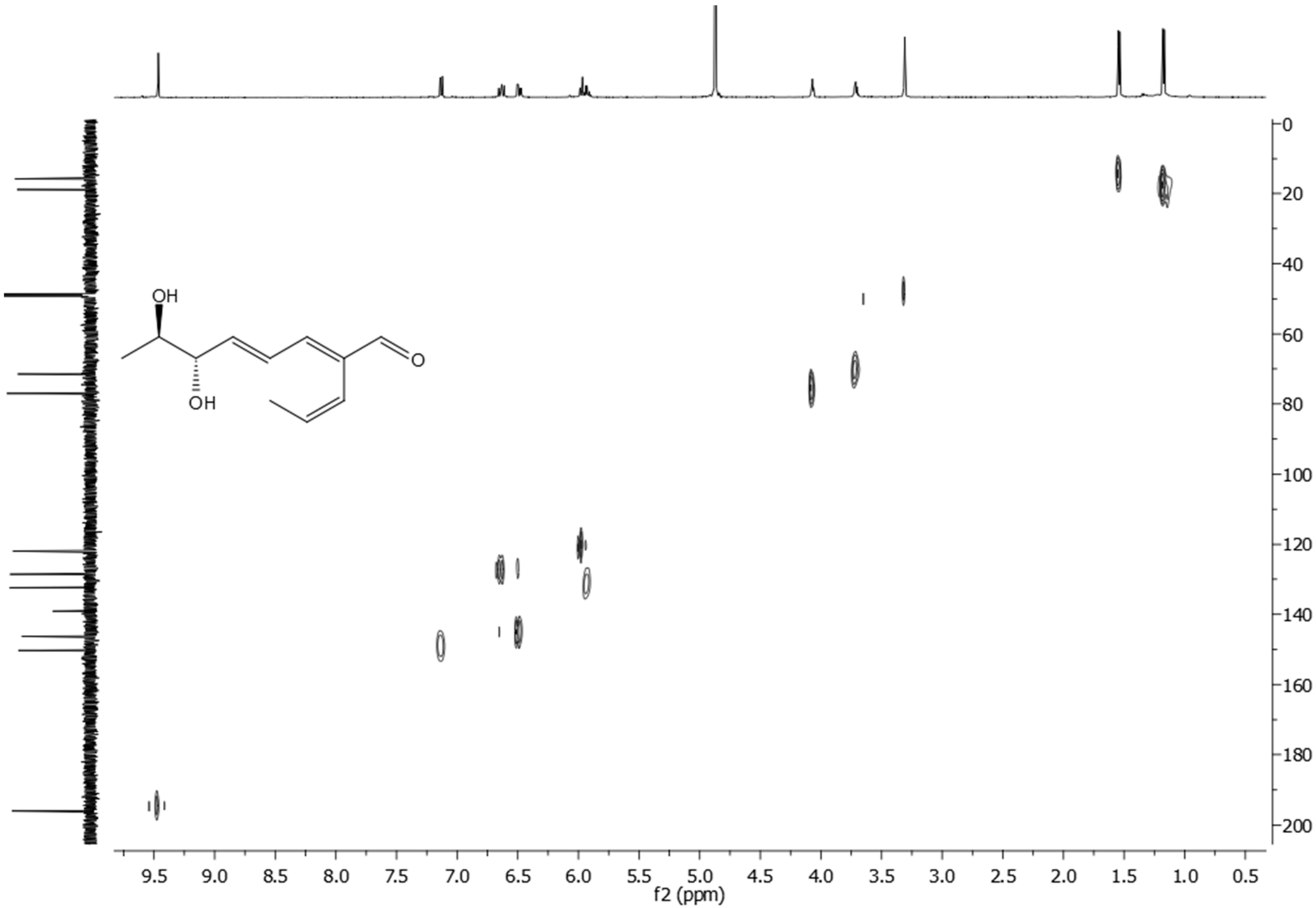

Figure S12. HSQC spectrum of talacyanol B (**2**).

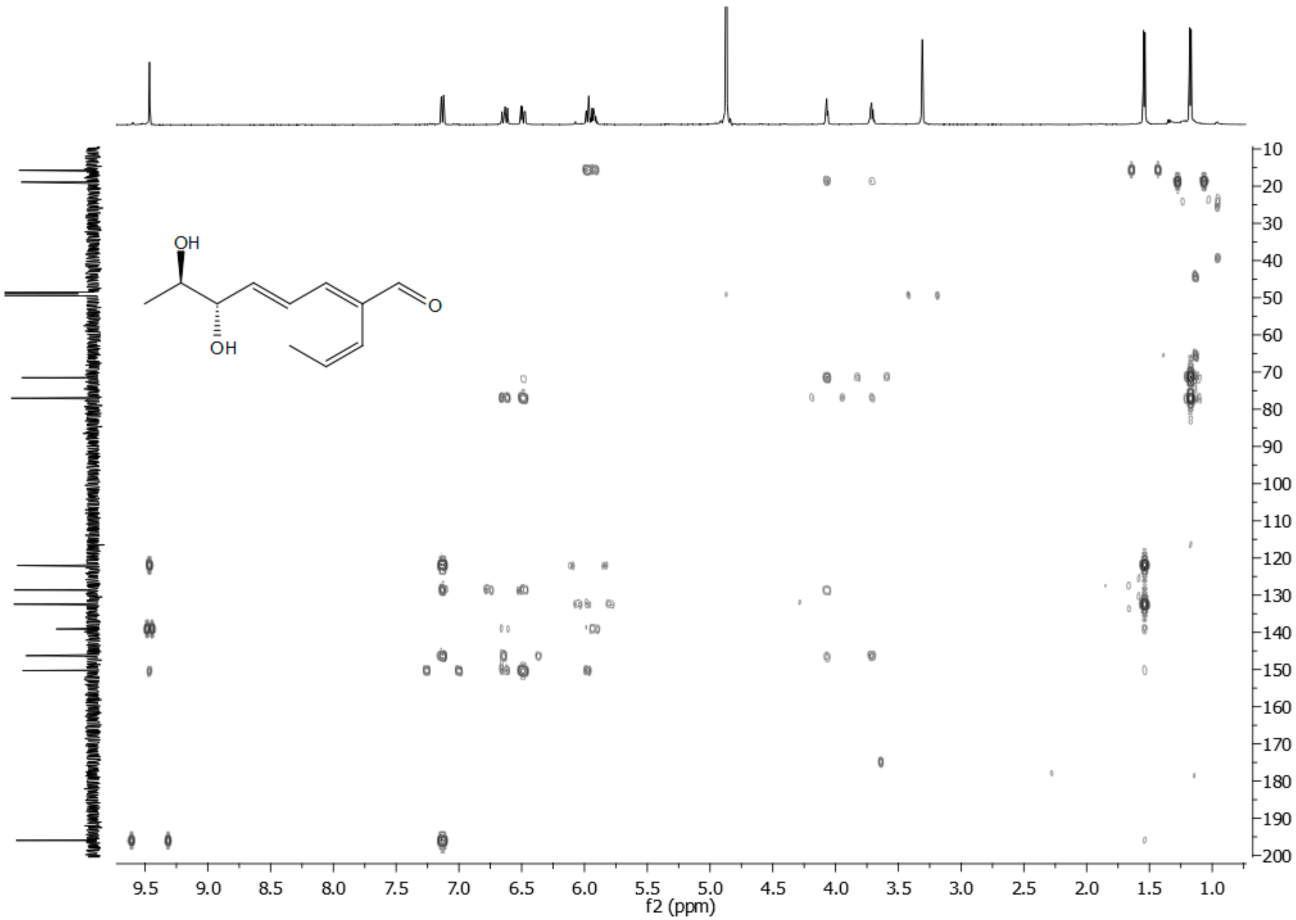

Figure S13. HMBC spectrum of talacyanol B (**2**).

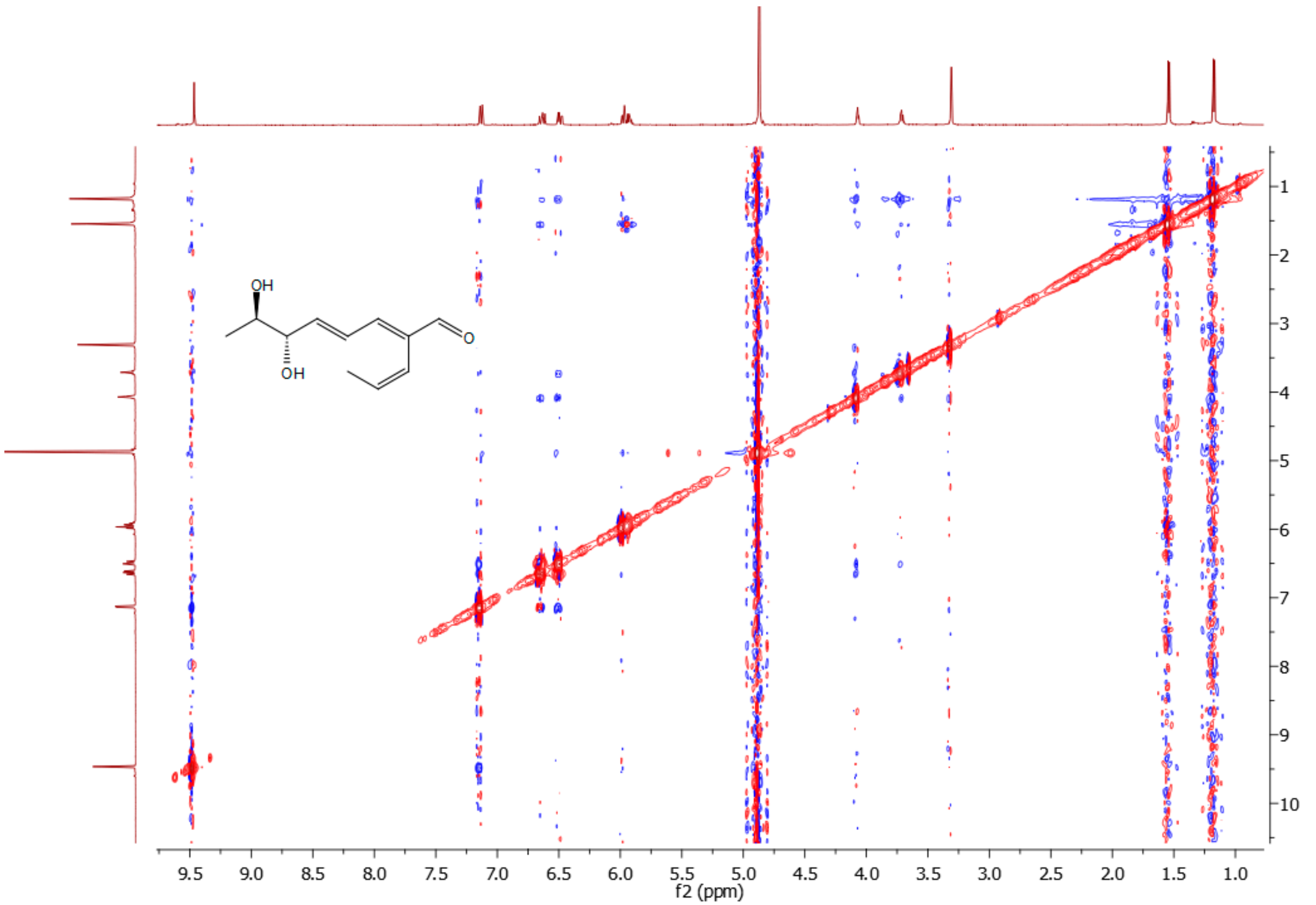

Figure S14. NOESY spectrum of talacyanol B (**2**).

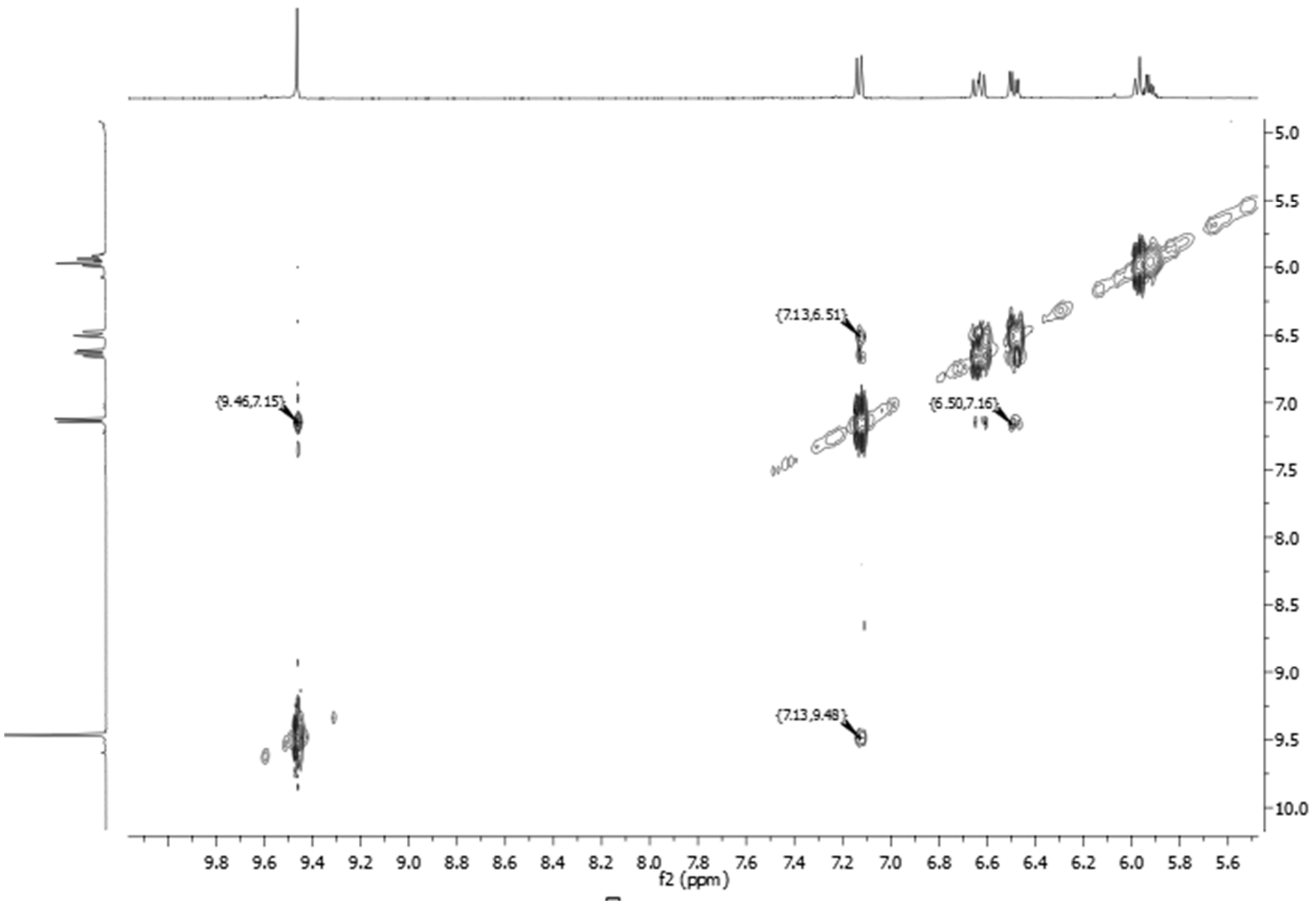

NOESY spectrum of **2** (zoomed).

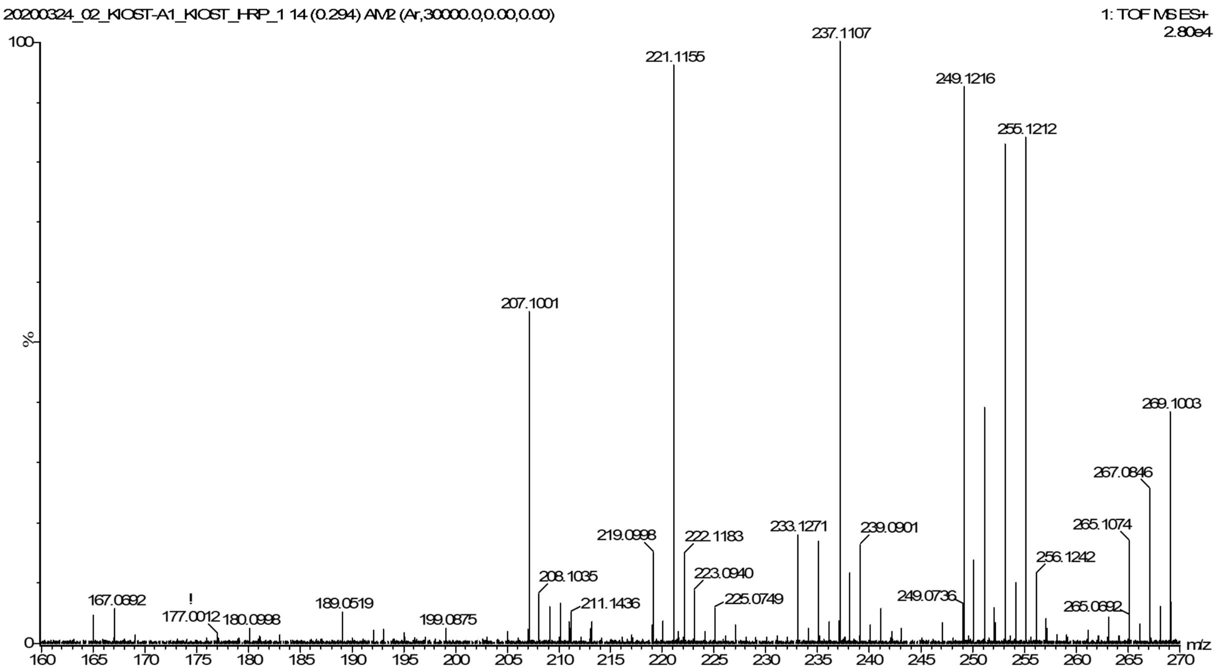

Monoisotopic Mass, Even Electron Ions
9 formula(e) evaluated with 1 results within limits (all results (up to 1000) for each mass)
Elements Used:
C: 0-15 H: 0-20 O: 0-3 Na: 0-1

Minimum:				-1.5				
Maximum:		500.0	5.0	100.0				
Mass	Calc. Mass	mDa	PPM	DBE	i-FIT	Norm	Conf(%)	Formula
221.1155	221.1148	0.1	0.5	2.5	677.0	n/a	n/a	C11 H18 O3 Na+

Figure S15. HR-ESIMS data of talacyanol C (**3**).

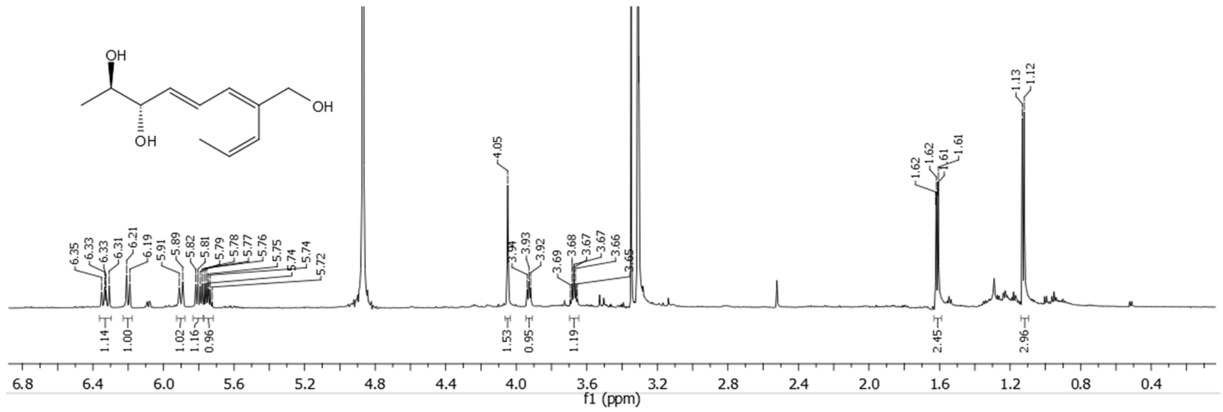

Figure S16. ^{1}H NMR (600 MHz, CD_3OD) spectrum of talacyanol C (**3**).

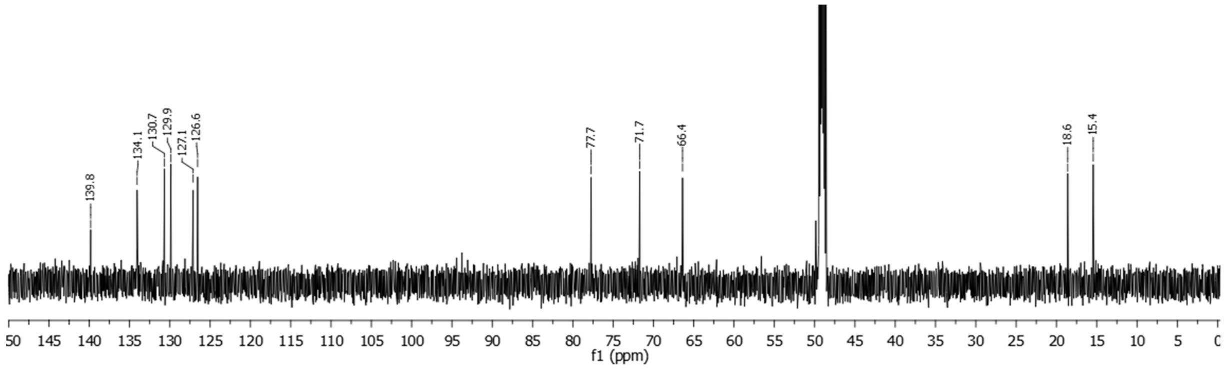

Figure S17. ^{13}C NMR (150 MHz, CD_3OD) spectrum of talacyanol C (**3**).

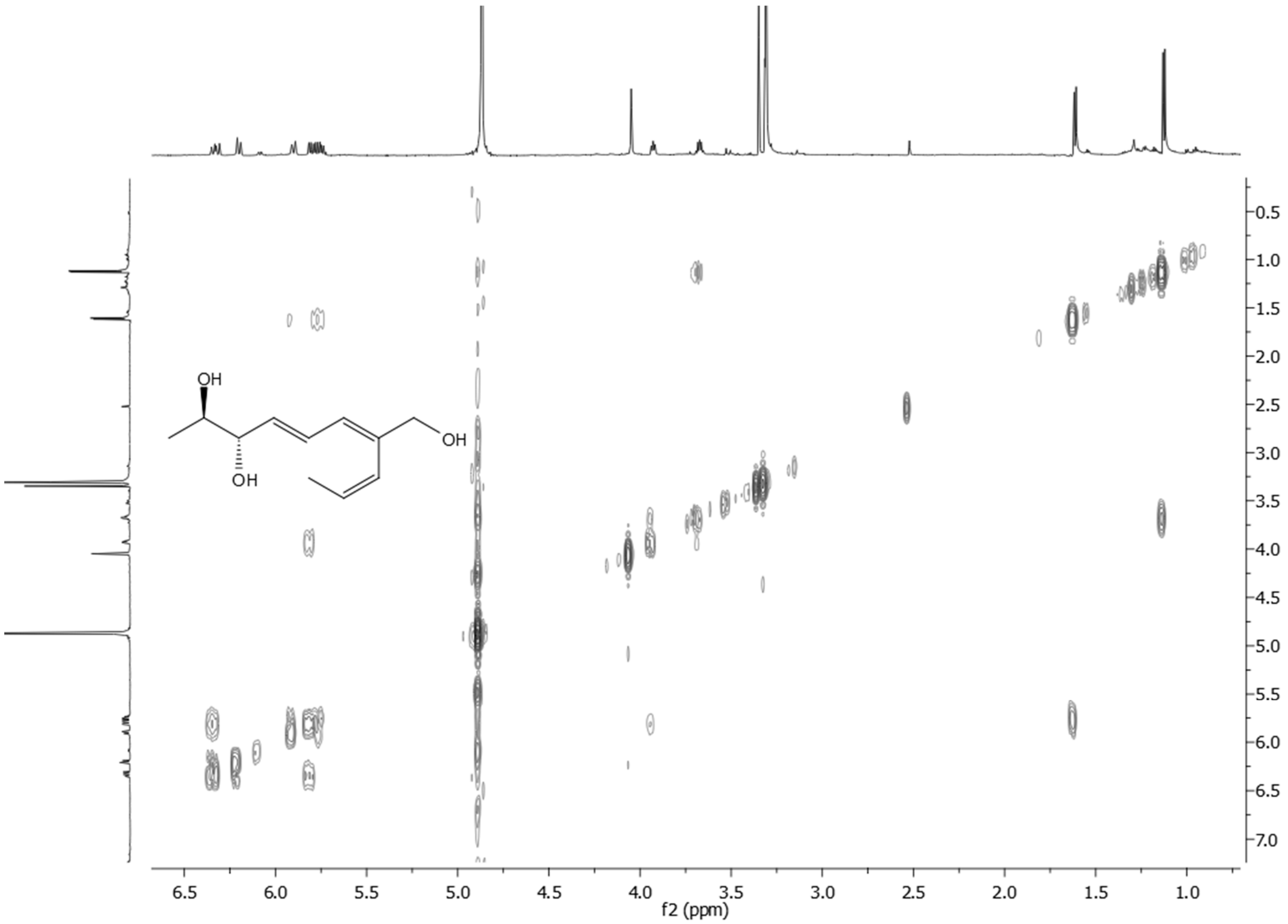

Figure S18. COSY spectrum of talacyanol C (**3**).

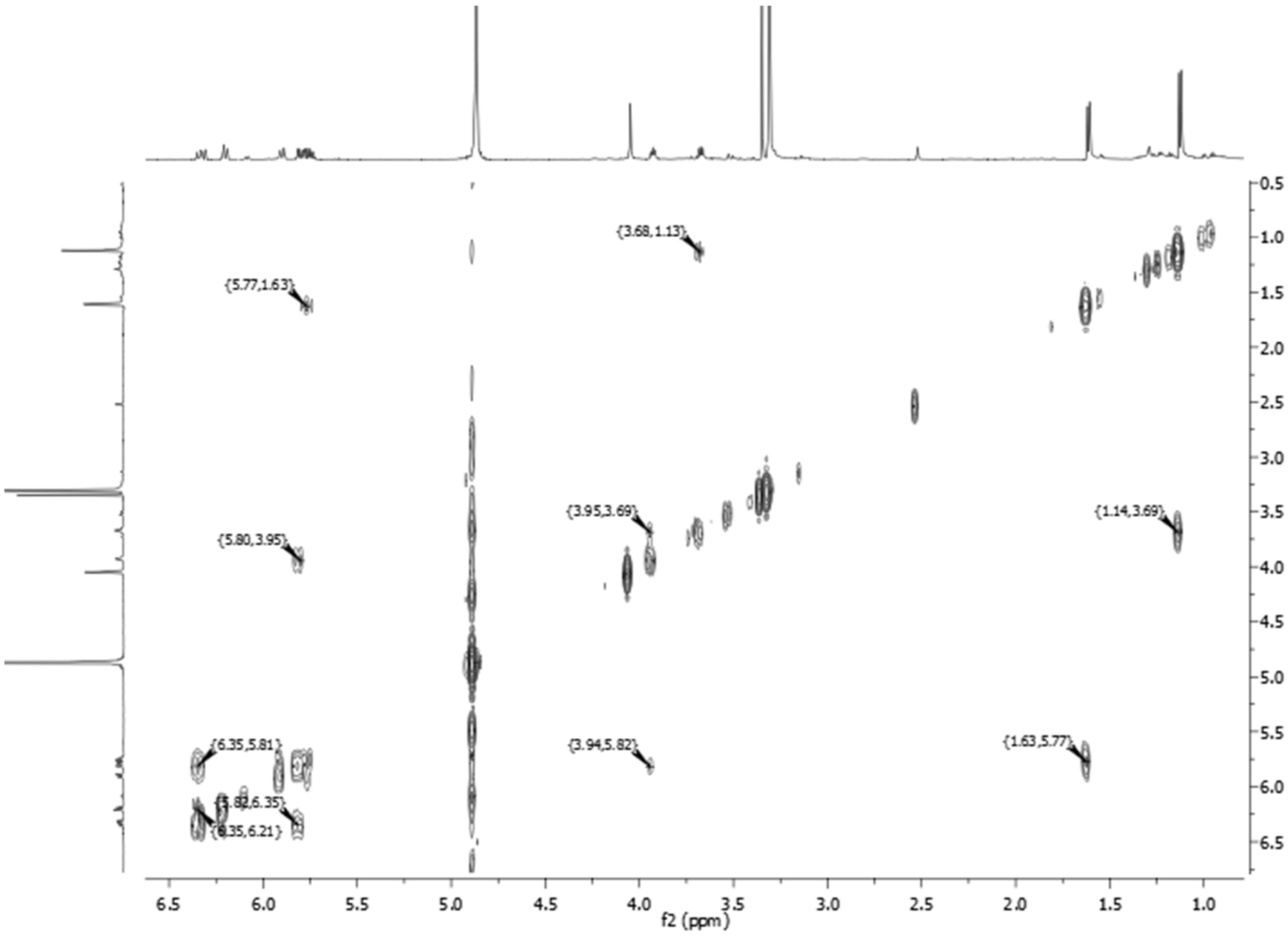

COSY spectrum of **3** (zoomed)

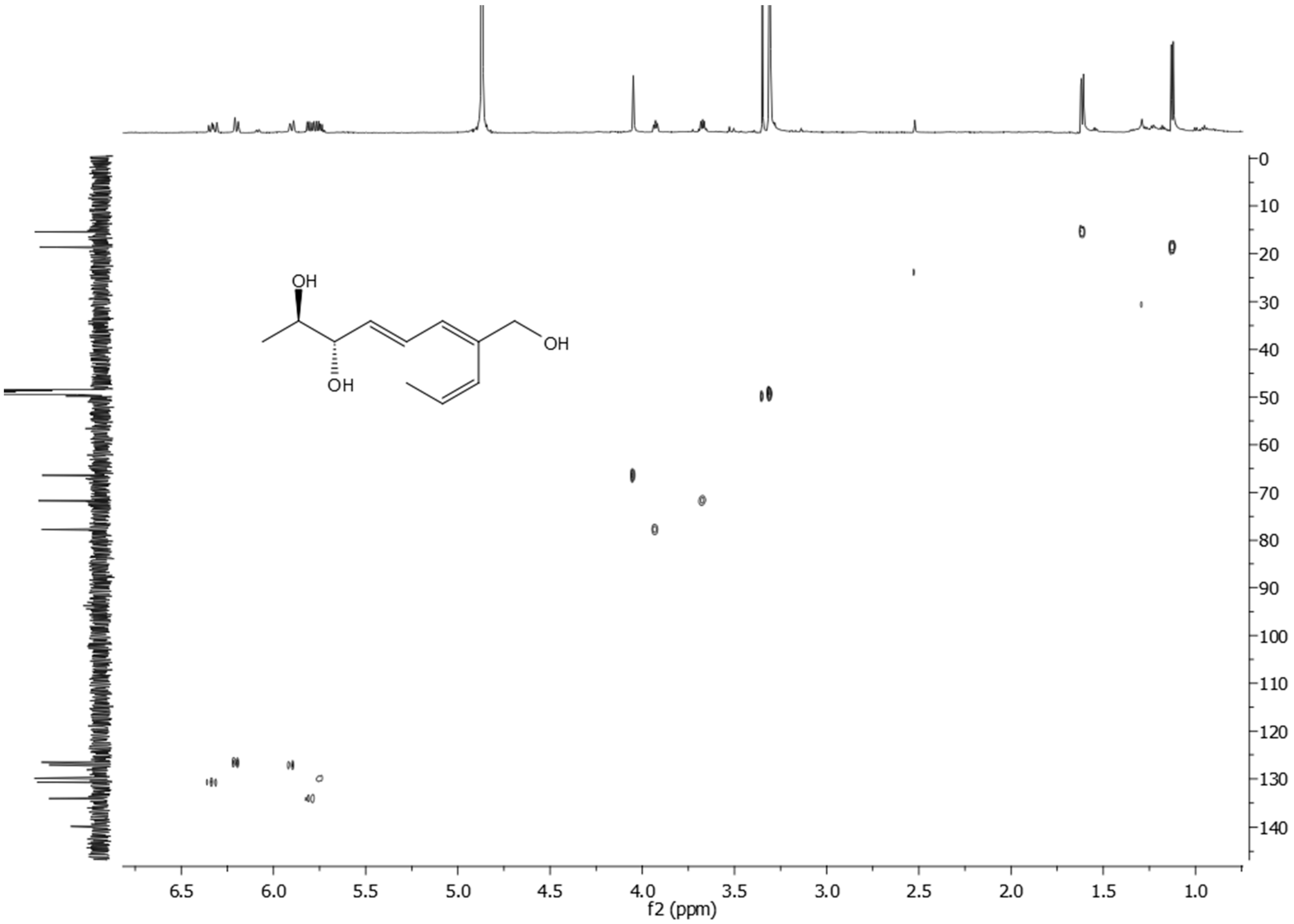

Figure S19. HSQC spectrum of talacyanol C (**3**).

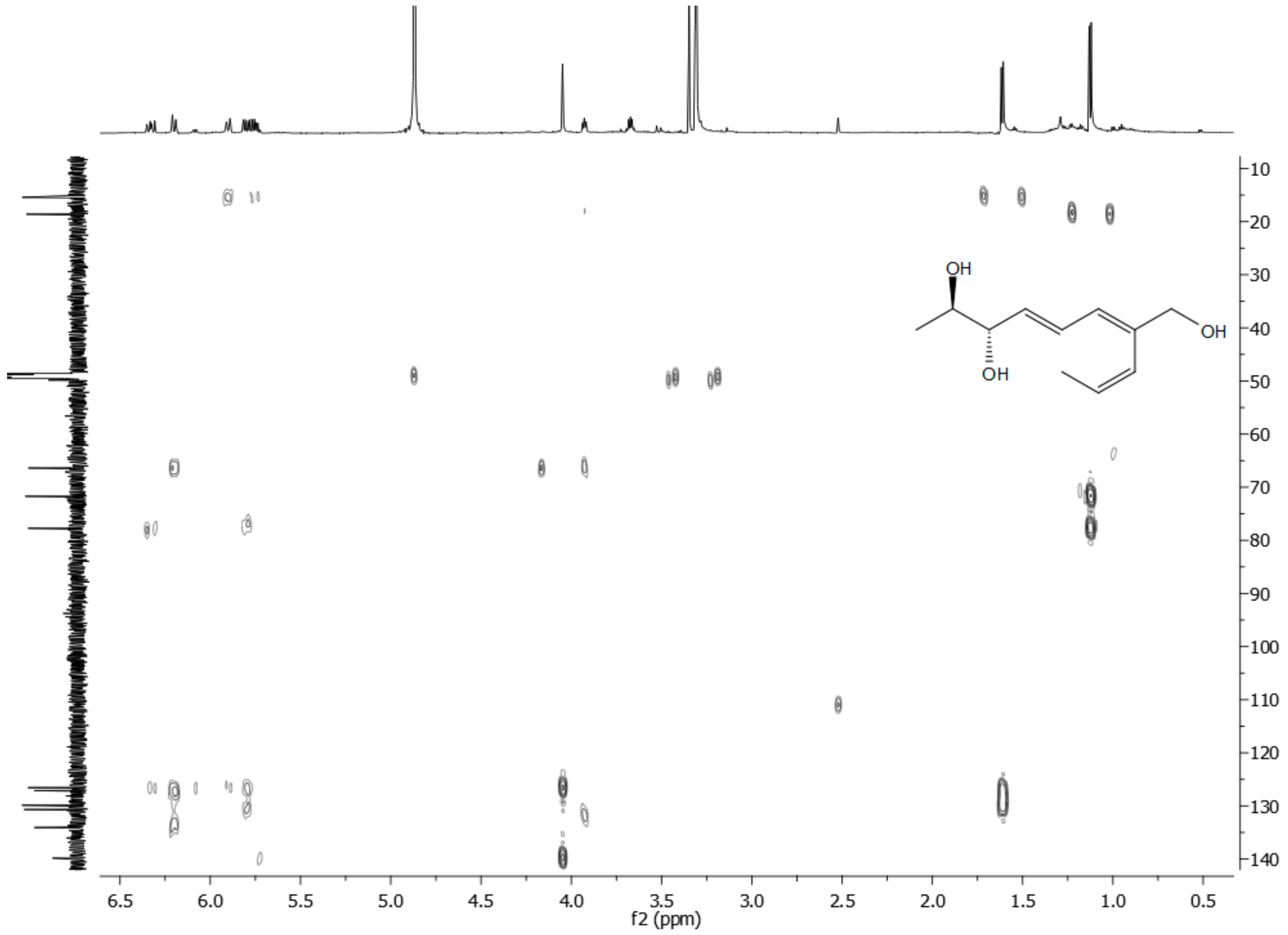

Figure S20. HMBC spectrum of talacyanol C (**3**).

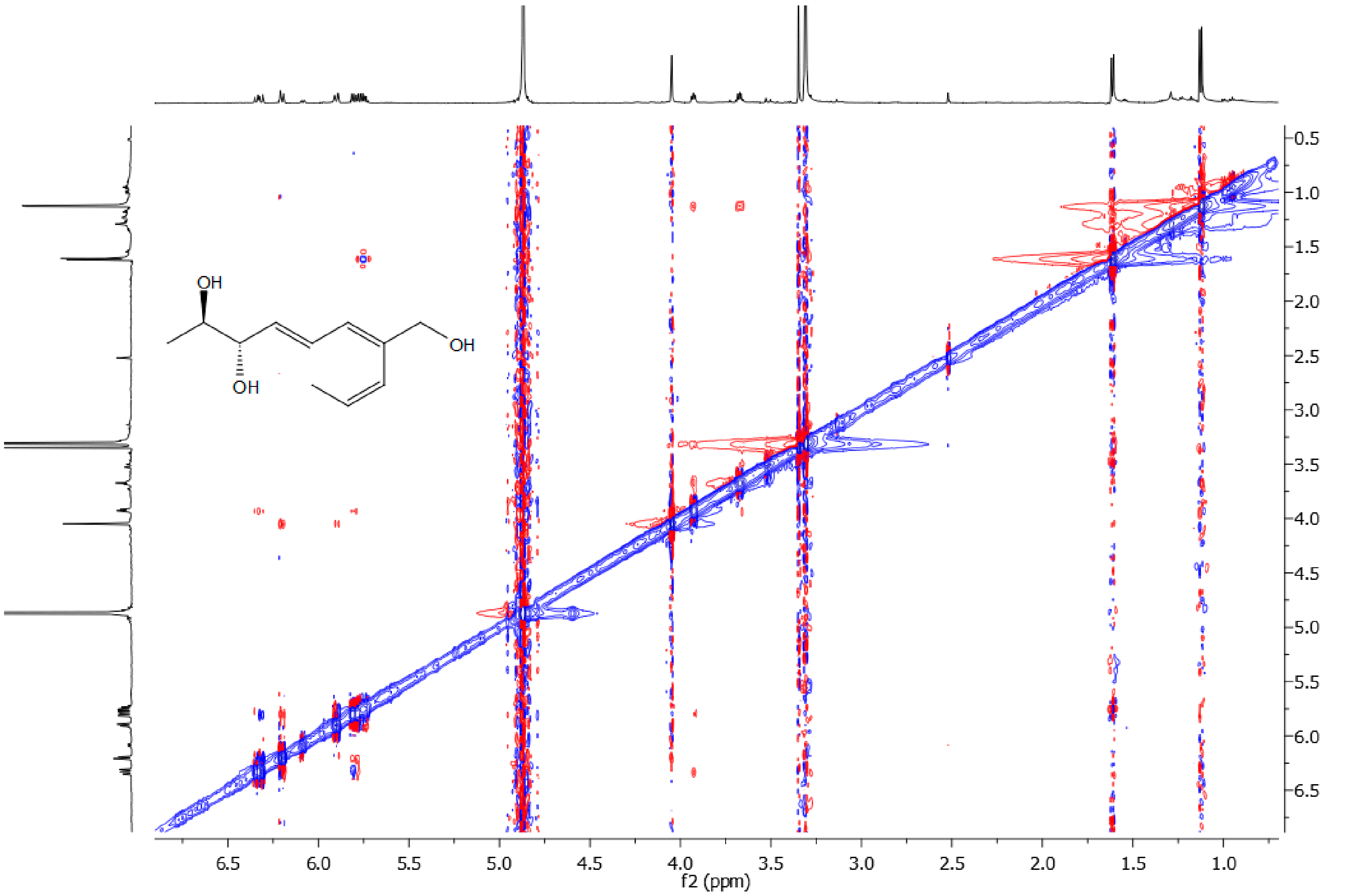

Figure S21. NOESY spectrum of talacyanol C (**3**).

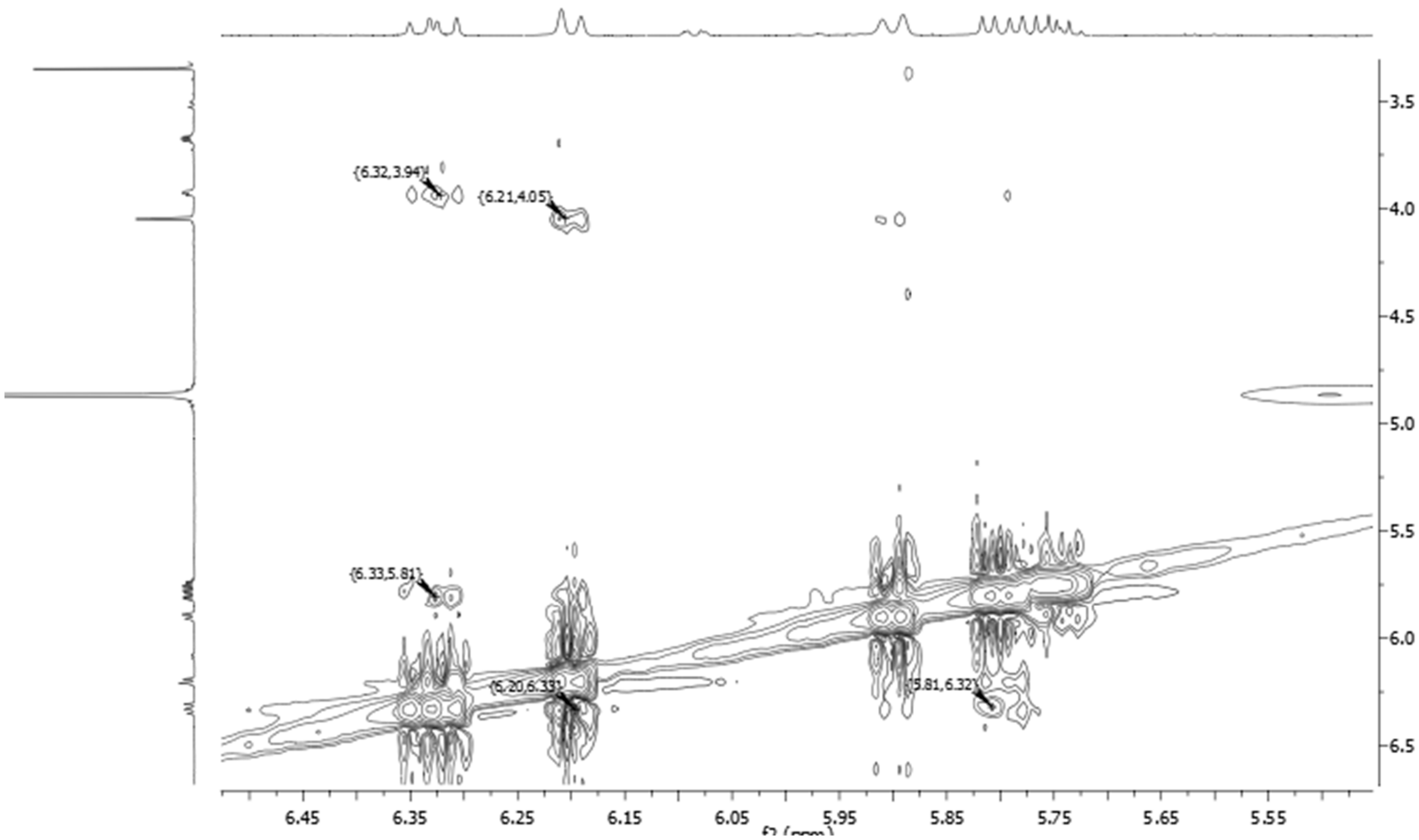

NOESY spectrum of **3** (zoomed).

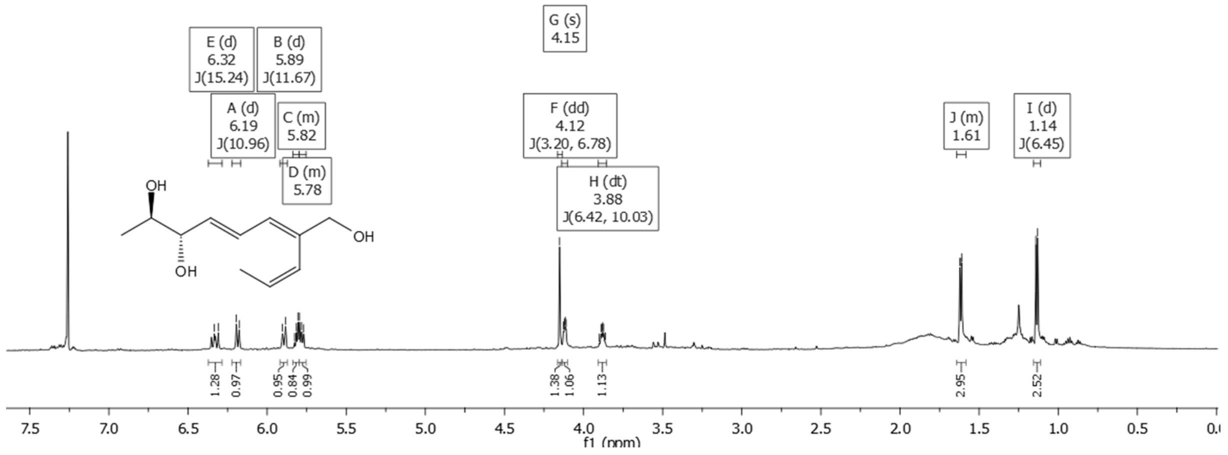

Figure S22. ^{1}H NMR (600 MHz, $CDCl_3$) spectrum of talacyanol C (**3**).

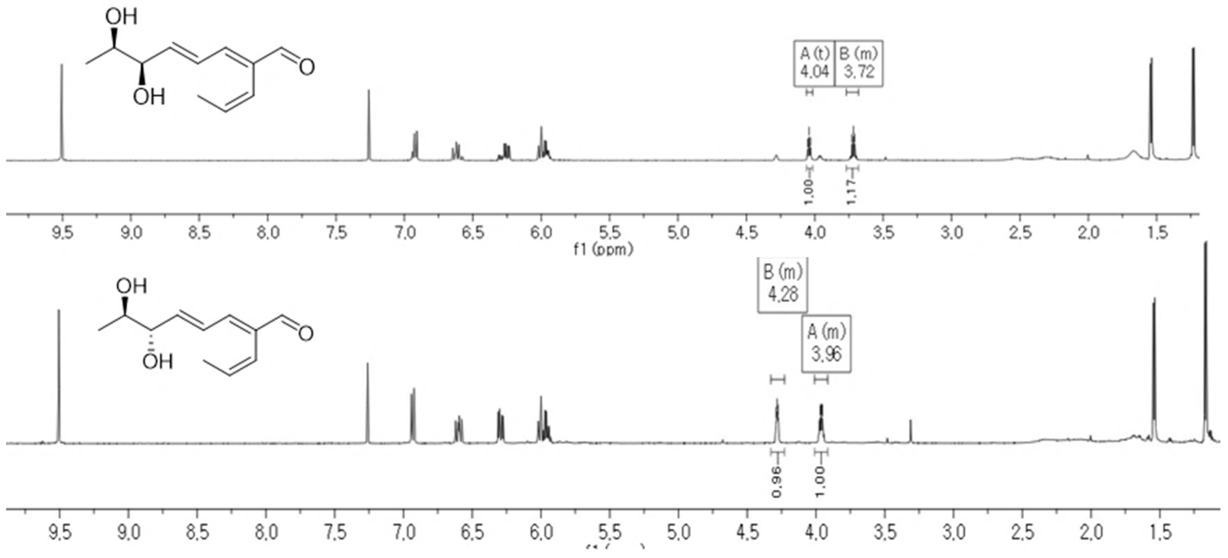

Figure S23. Comparision of ^{1}H NMR (600 MHz, $CDCl_3$) spectra between talacyanols A (**1**) and B (**2**).

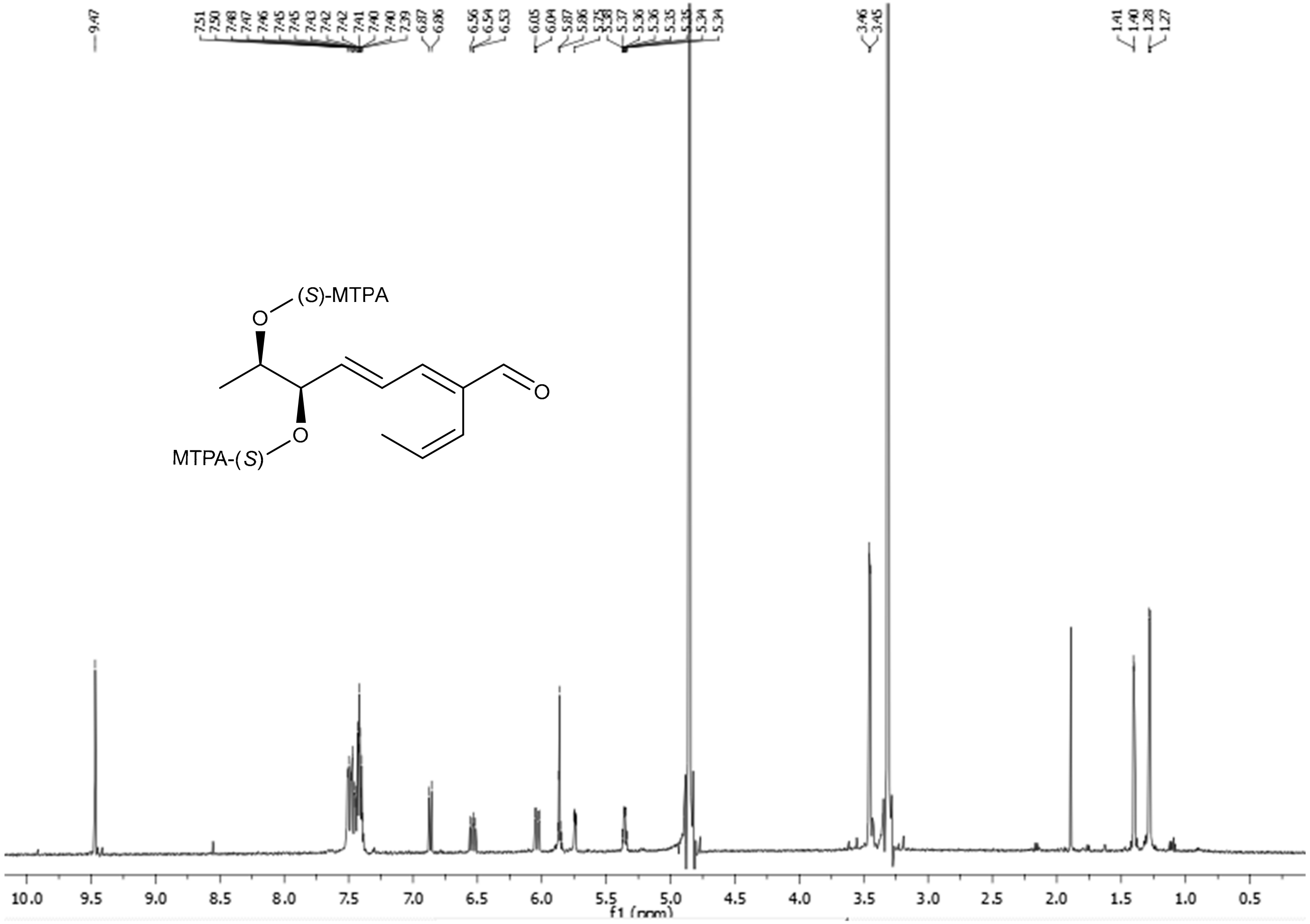

Figure S24. ^{1}H NMR (600 MHz, CD_3OD) spectrum of bis-(*S*)-MTPA ester of **1** (**1a**).

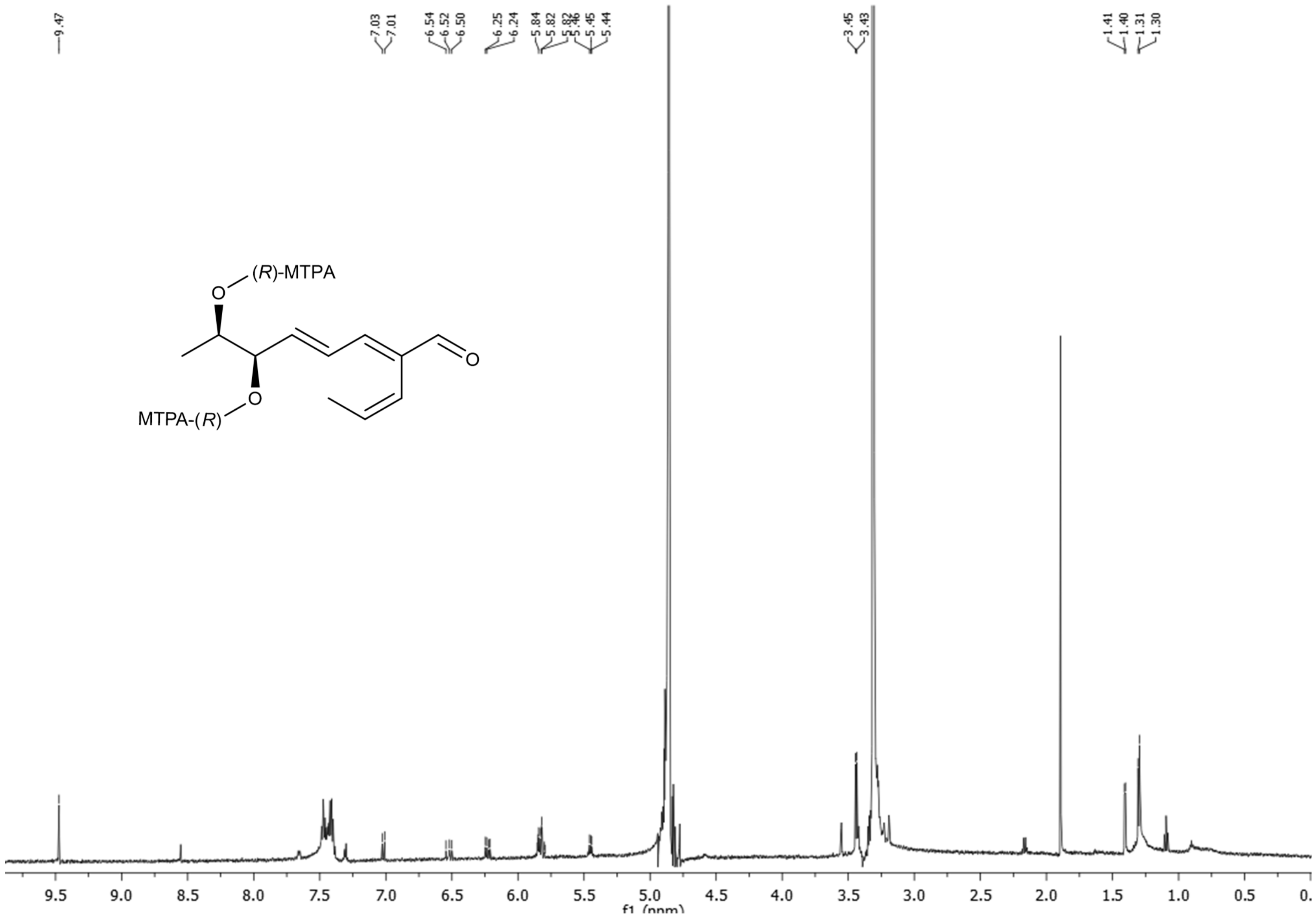

Figure S25. ^{1}H NMR (600 MHz, CD_3OD) spectrum of bis-(*R*)-MTPA ester of **1** (**1b**)

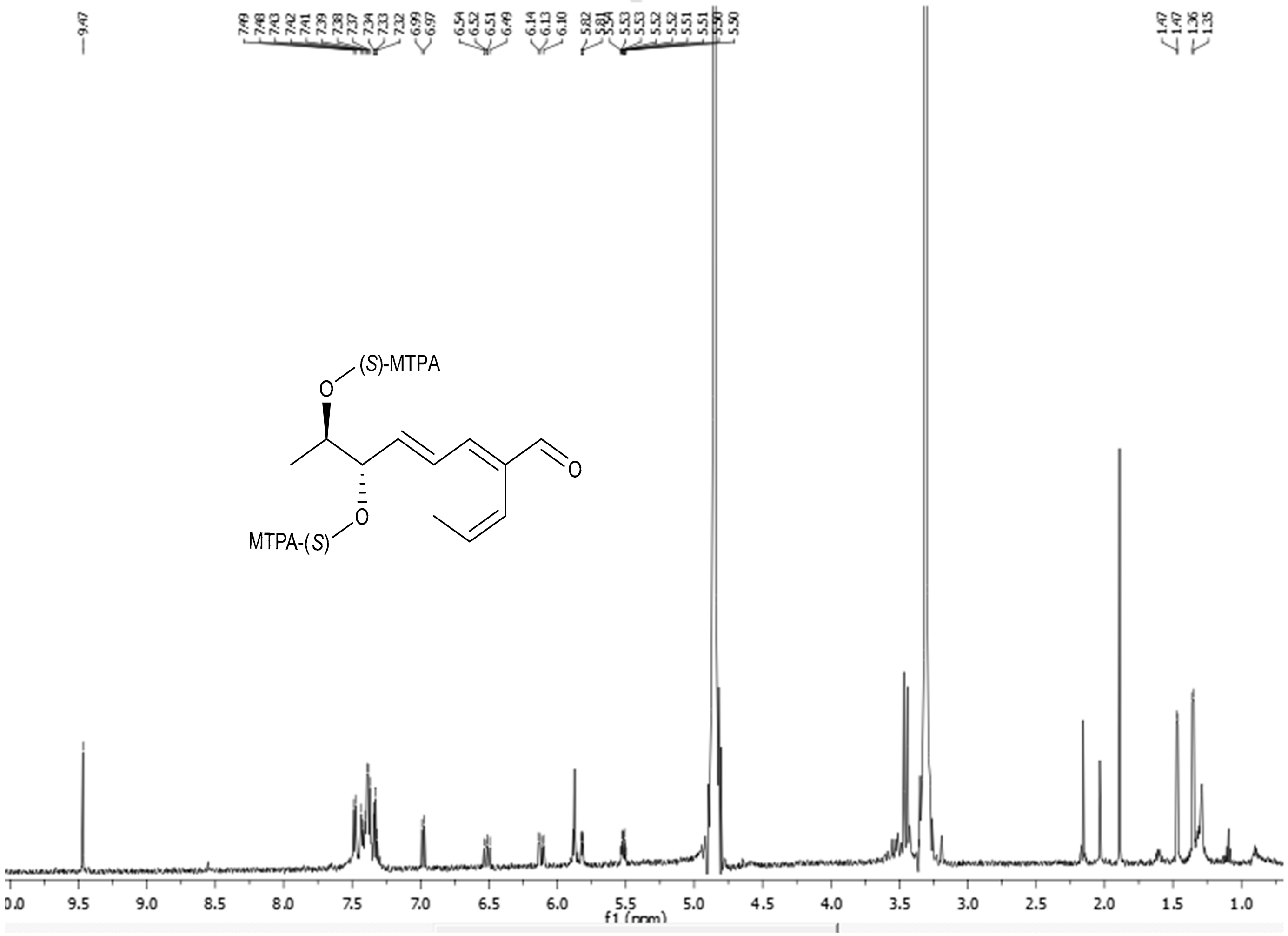

Figure S26. ^{1}H NMR (600 MHz, CD_3OD) spectrum of bis-(*S*)-MTPA ester of **2** (**2a**).

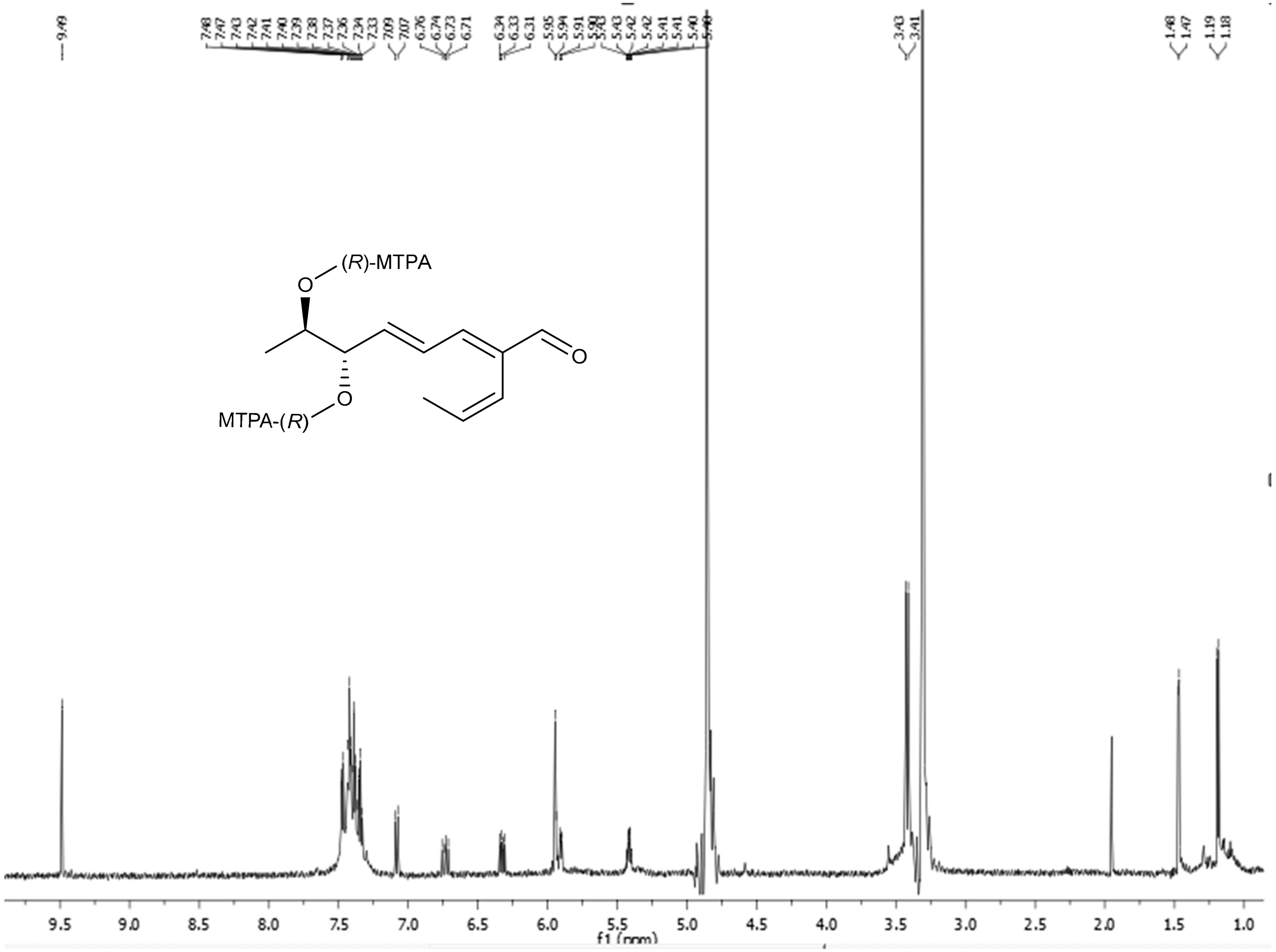

Figure S27. ^1H NMR (600 MHz, CD_3OD) spectrum of bis-(*R*)-MTPA ester of **2** (**2b**).

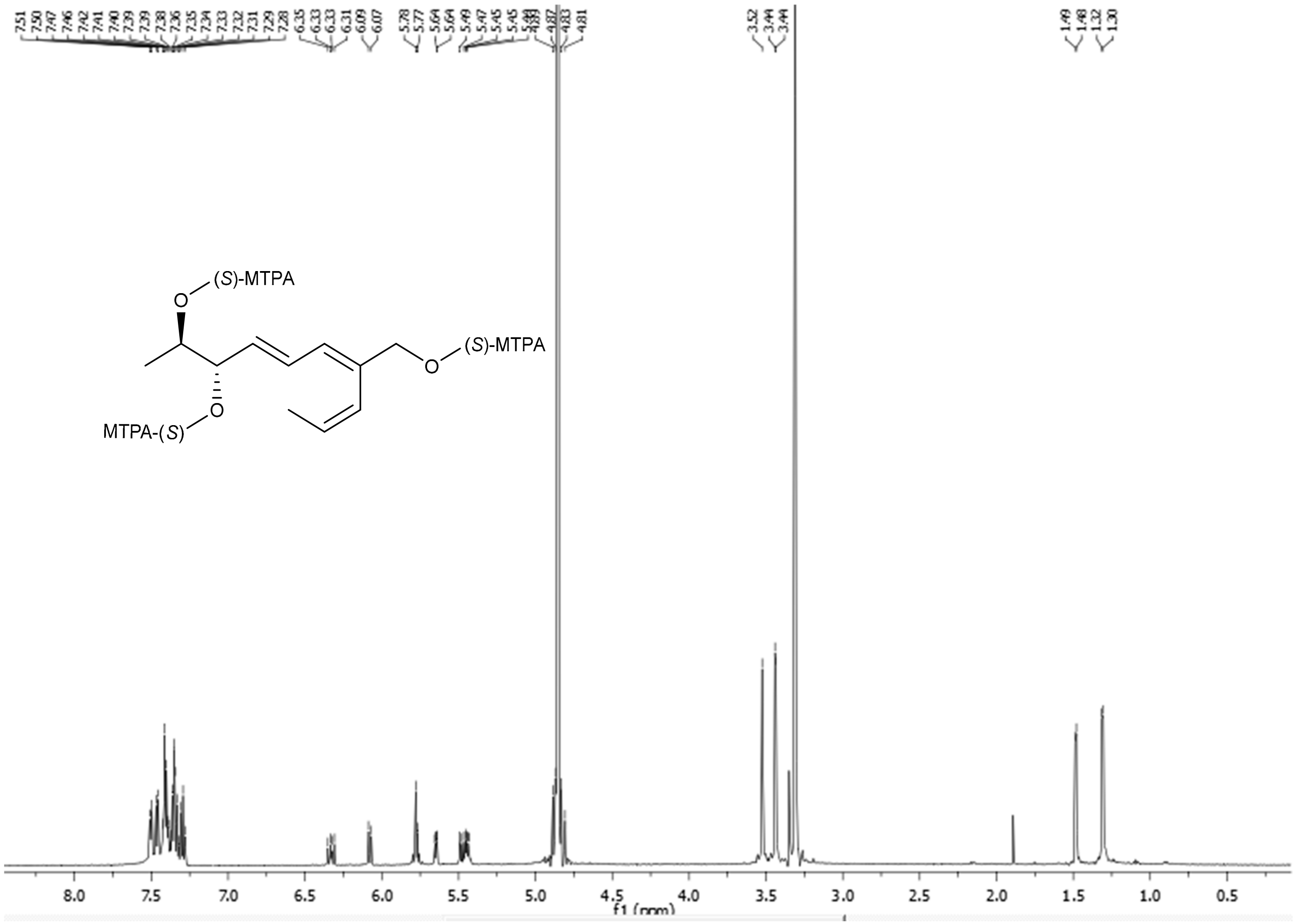

Figure S28. ^{1}H NMR (600 MHz, CD_3OD) spectrum of tri-(*S*)-MTPA ester of **3** (**3a**).

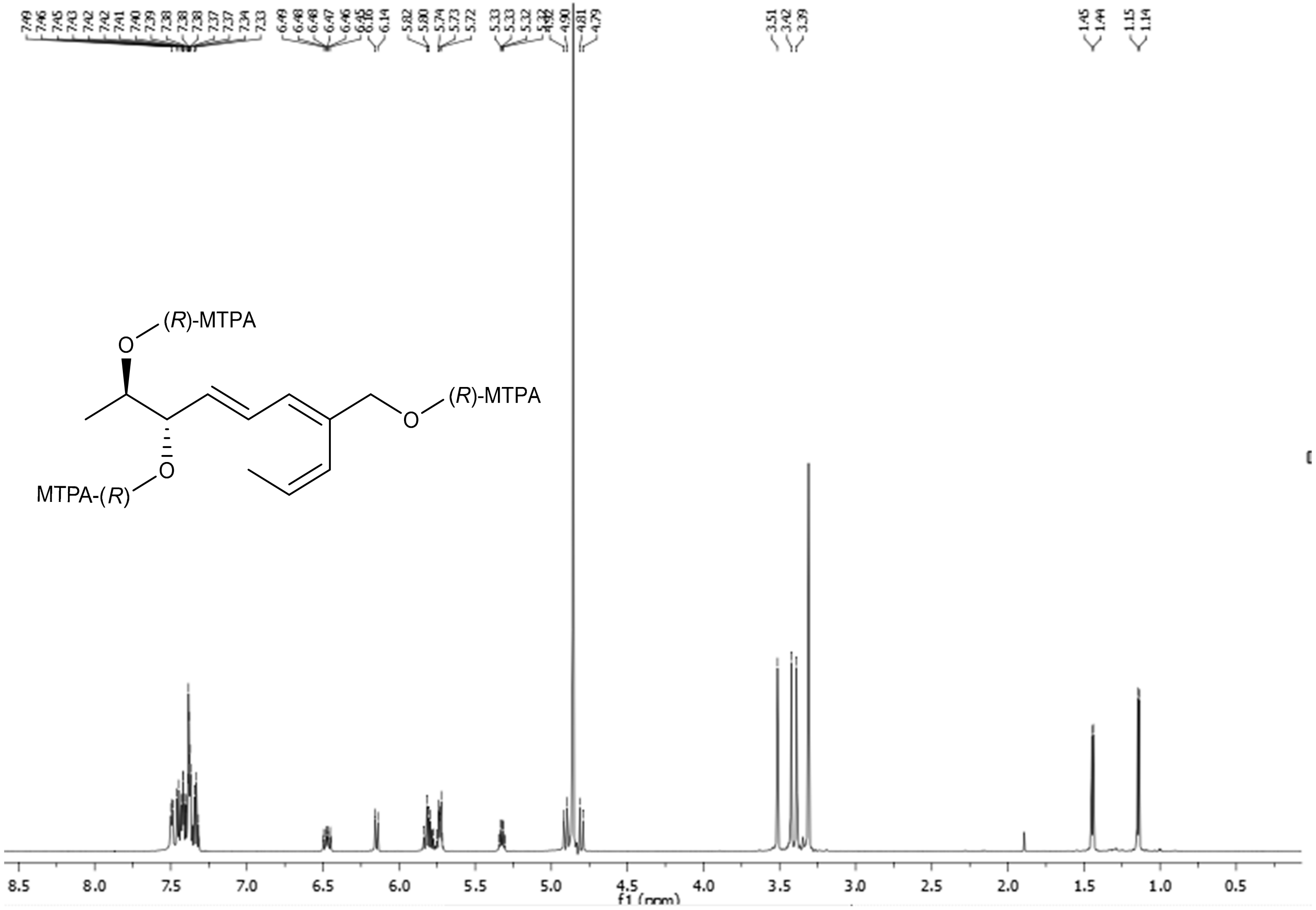

Figure S29. ^{1}H NMR (600 MHz, CD_3OD) spectrum of tri-(*R*)-MTPA ester of **3** (**3b**).

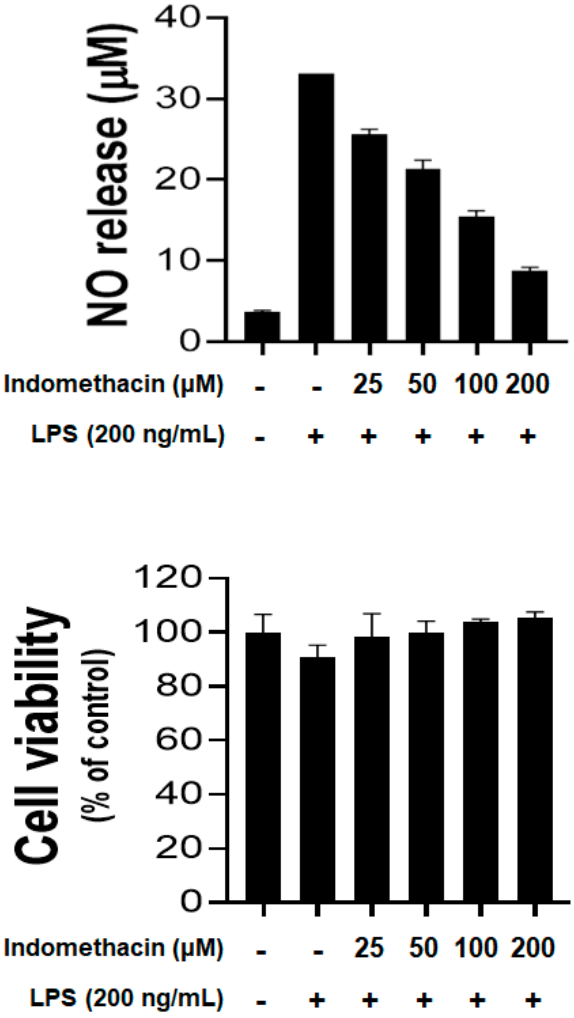

Figure S30. NO release level with 25~200 μM of indomethacin as a positive control.

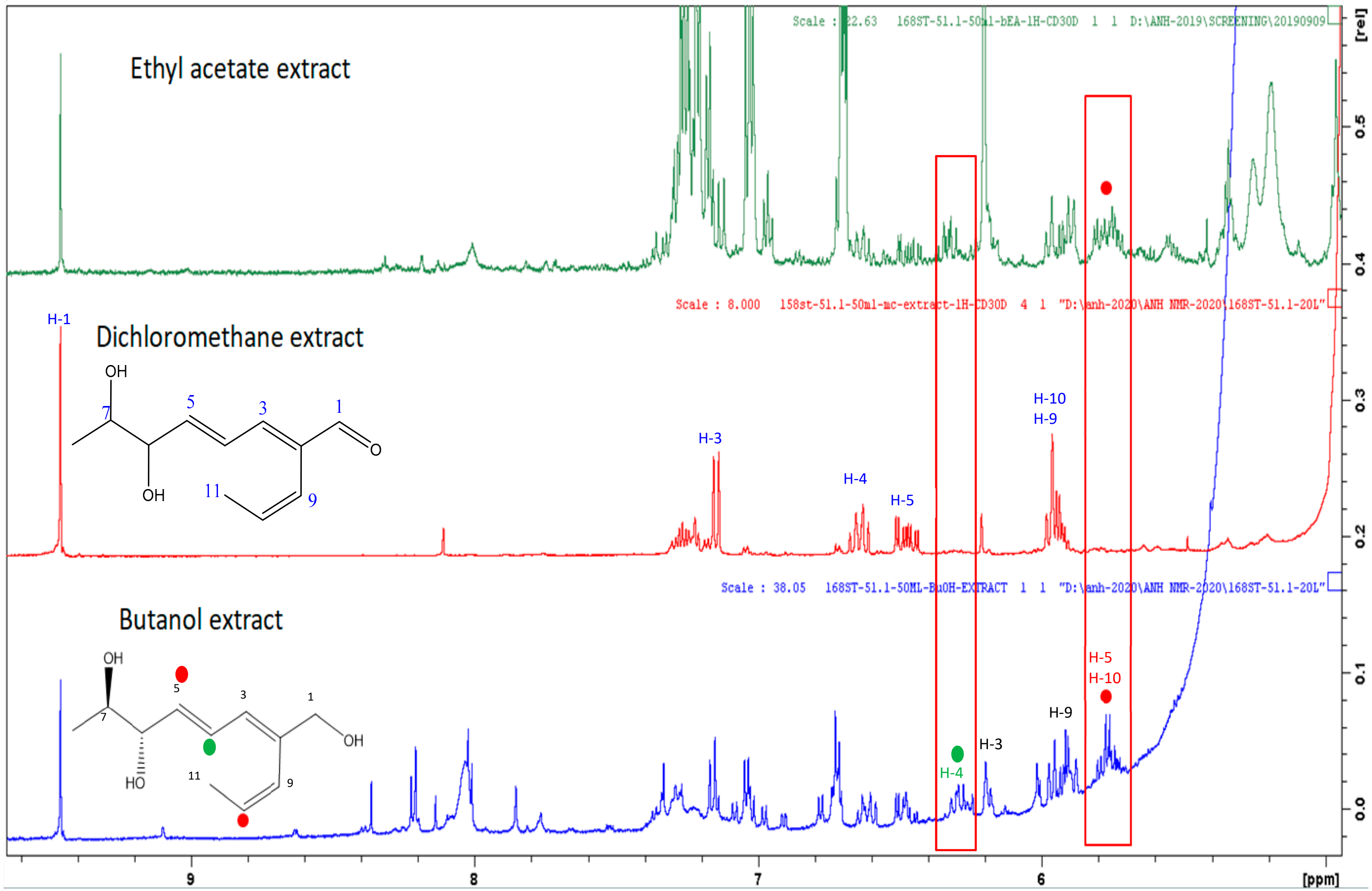

Figure S31. Comparision of ^{1}H NMR (600 MHz, CD_3OD) spectra between ethyl acetate, dichloromethane, and butanol extracts containing compound **3**.

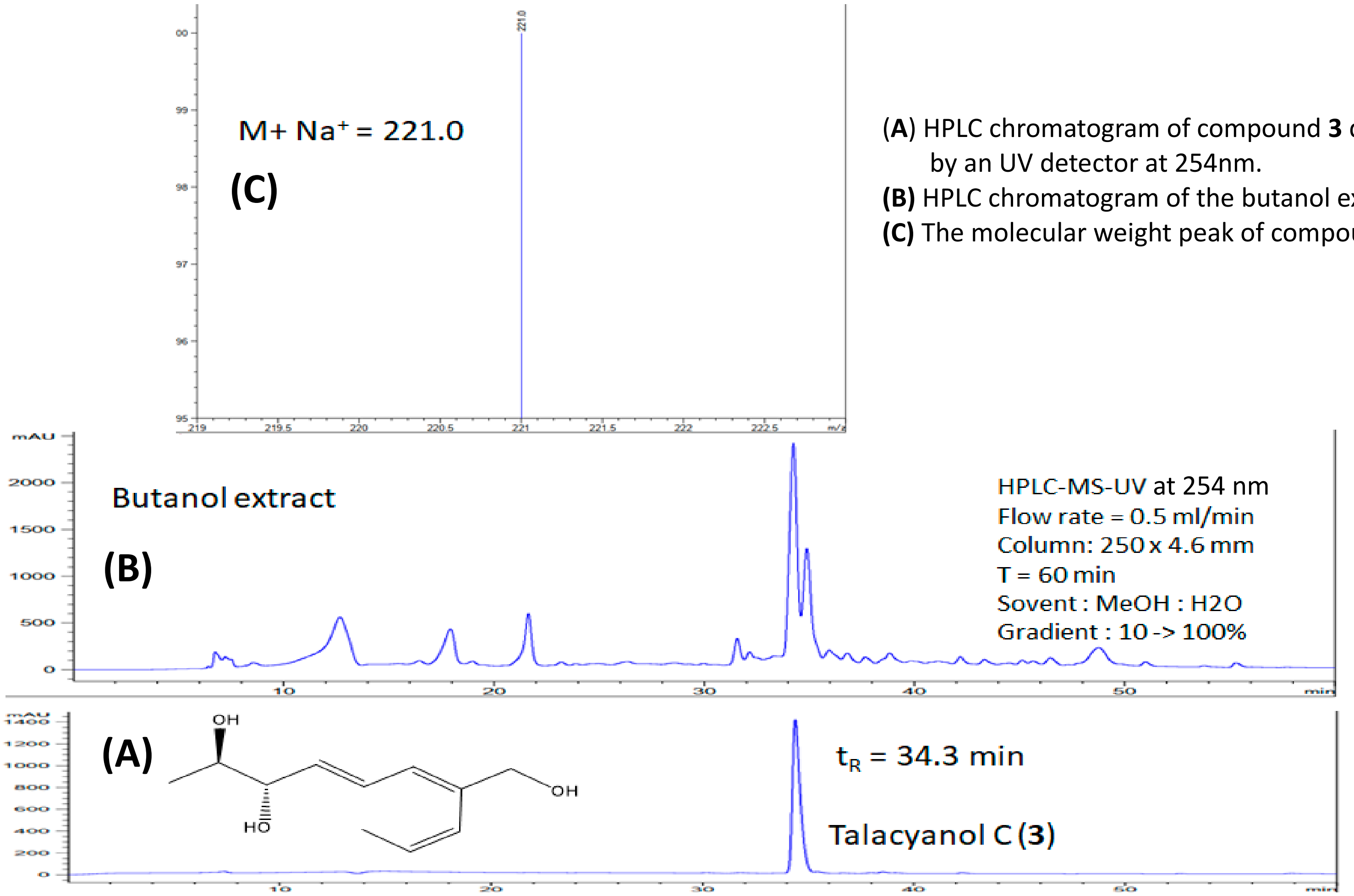

(**A**) HPLC chromatogram of compound **3** detected by an UV detector at 254nm.
(B) HPLC chromatogram of the butanol extract.
(C) The molecular weight peak of compound **3** detected at 34.3 min.

Figure S32. Detection of talacyanol C (**3**) in the butanol extract by HPLC-MS-UV.

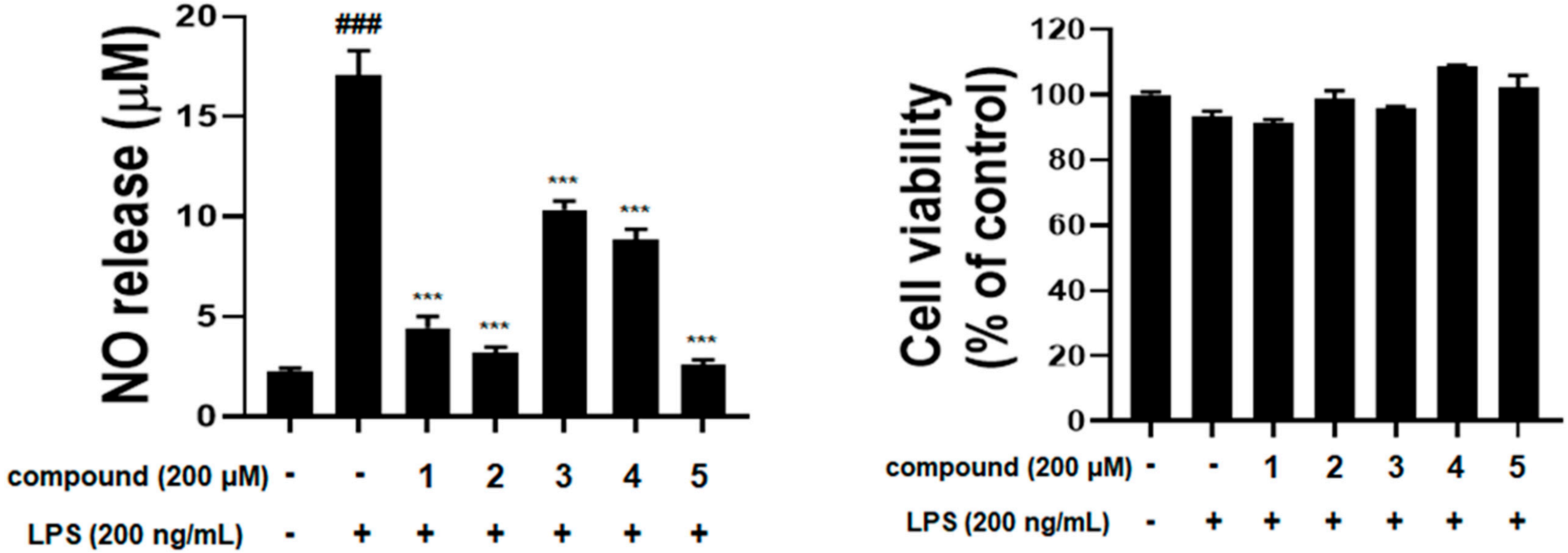

Figure S33. The inhibitory effect on NO production of compounds **1**-**5** at a concentration of 200 μM.
